# IFN-λ and microRNAs are important modulators of the pulmonary innate immune response against influenza A (H1N2) infection in pigs

**DOI:** 10.1371/journal.pone.0194765

**Published:** 2018-04-20

**Authors:** Louise Brogaard, Lars E. Larsen, Peter M. H. Heegaard, Christian Anthon, Jan Gorodkin, Ralf Dürrwald, Kerstin Skovgaard

**Affiliations:** 1 Section for Protein Science and Signaling Biology, Department of Biotechnology and Biomedicine, Technical University of Denmark, Kongens Lyngby, Denmark; 2 Division of Diagnostics and Scientific Advice–Virology, National Veterinary Institute, Technical University of Denmark, Kongens Lyngby, Denmark; 3 Center for non-coding RNA in Technology and Health (RTH), Department of Veterinary and Animal Science, University of Copenhagen, Frederiksberg, Denmark; 4 Department of Infectious Diseases, Robert Koch Institute, Berlin, Germany; The Ohio State University, UNITED STATES

## Abstract

The innate immune system is paramount in the response to and clearance of influenza A virus (IAV) infection in non-immune individuals. Known factors include type I and III interferons and antiviral pathogen recognition receptors, and the cascades of antiviral and pro- and anti-inflammatory gene expression they induce. MicroRNAs (miRNAs) are increasingly recognized to participate in post-transcriptional modulation of these responses, but the temporal dynamics of how these players of the antiviral innate immune response collaborate to combat infection remain poorly characterized. We quantified the expression of miRNAs and protein coding genes in the lungs of pigs 1, 3, and 14 days after challenge with swine IAV (H1N2). Through RT-qPCR we observed a 400-fold relative increase in IFN-λ3 gene expression on day 1 after challenge, and a strong interferon-mediated antiviral response was observed on days 1 and 3 accompanied by up-regulation of genes related to the pro-inflammatory response and apoptosis. Using small RNA sequencing and qPCR validation we found 27 miRNAs that were differentially expressed after challenge, with the highest number of regulated miRNAs observed on day 3. In contrast, the number of protein coding genes found to be regulated due to IAV infection peaked on day 1. Pulmonary miRNAs may thus be aimed at fine-tuning the initial rapid inflammatory response after IAV infection. Specifically, we found five miRNAs (ssc-miR-15a, ssc-miR-18a, ssc-miR-21, ssc-miR-29b, and hsa-miR-590-3p)–four known porcine miRNAs and one novel porcine miRNA candidate–to be potential modulators of viral pathogen recognition and apoptosis. A total of 11 miRNAs remained differentially expressed 14 days after challenge, at which point the infection had cleared. In conclusion, the results suggested a role for miRNAs both during acute infection as well as later, with the potential to influence lung homeostasis and susceptibility to secondary infections in the lungs of pigs after IAV infection.

## Introduction

Influenza A is an RNA virus of the *Orthomyxoviridae* family with a single-stranded, negative sense segmented genome causing highly contagious respiratory infections in many species, including humans and pigs. Seasonal influenza in humans is a self-limiting disease, and healthy individuals usually recover within a week. However, in vulnerable individuals, such as the elderly, the immunocompromised, or individuals with chronic low-grade inflammation, the course of disease may be more severe, with a higher incidence of secondary infections and fatalities [1]. New variant viruses emerge continuously due to accumulation of mutations in the viral genome during replication. Due to these continuous antigenic changes, immunity that is acquired during one influenza season (either by natural infection or vaccination) often provide insufficient protection against the strains that dominate the following seasons [2]. Control of the IAV infection is therefore highly dependent on an efficient innate immune response [3].

It has been documented that IAV infection induces a rapid interferon response dominated by type I interferons (IFN-α, IFN-β) and the more recently described type III interferons (IFN-λ) [4,5]. Interferons are responsible for the induction of a multitude of genes termed interferon-stimulated genes (ISGs), including PKR, Mx1, IFITMs, and ISG15. These ISGs establish an antiviral state within the cell that effectively combats the infection and spreads to neighboring cells via secreted interferon [5]. This innate antiviral response is initiated by pathogen recognition receptors (PRRs) that recognize viral RNA, such as the cytoplasmic Toll-like receptors (TLRs) 3 and 7 and the RIG-I-like receptors (RLRs) RIG-I and MDA5 [3]. Upon PRR recognition of IAV, adapter proteins such as MyD88, MAVS, and TRIF initiate signaling cascades that ultimately lead to the production of cytokines such as IL-1, IL-6, and TNF, as well as chemokines IL-8, CXCL2, and CXCL10 that recruit monocytes and macrophages to the infected tissue [6–8].

The pig has proven to be a valid model for human IAV infection as it displays many relevant characteristics for human IAV infection. The pathogenesis of IAV infection is highly similar in pigs and humans [9,10], pigs are natural hosts for IAV and carry an IAV receptor distribution which is similar to humans’ [11–13], they have extensive immunological and physiological similarities to humans [14–16], and pigs present clinical signs upon IAV infection that are comparable to humans’ [9,17]. Many aspects of the porcine local pulmonary tissue response are however still unresolved. Particularly, there is a lack of knowledge regarding the role of microRNA (miRNA) involvement in the innate immune response against viral infections. Since their discovery in the early 1990ies, miRNAs have been demonstrated to be associated with a multitude of cellular and developmental processes [18–20]. miRNAs are a class of short (~22 nt) non-coding RNA molecules that regulate translation via antisense complementarity to target mRNA transcripts. miRNA-mRNA interaction will typically lead to destabilization and degradation of the mRNA target, thereby effectively inhibiting the protein output [21,22].

A vast range of pathologies have been found to induce changes in host miRNA expression, including many viral infections [23,24]. This has prompted research into the field of therapeutic and biomarker potential for miRNAs in viral infections and many other diseases. In recent years, several studies describing miRNA expression during IAV infection have emerged. Among these are studies in both animal [25,26] and *in vitro* [27–29] models demonstrating that different IAV strains and subtypes of varying virulence induce distinct miRNA responses in the host. Given the potential importance of miRNAs in the modulation of inflammatory responses [30,31], miRNAs are likely to play an important role in the refinement of the innate immune defense against IAV.

Investigations into miRNA regulation of innate immune processes have provided experimental evidence for a large number of miRNA-target interactions [32]. A number of *in silico* resources for miRNA target prediction are likewise available [33]. Combined, the large number of miRNAs that are experimentally validated or computationally predicted to target immune-related transcripts yields a long list of miRNAs for which the function in relation to IAV infection is important to clarify. One miRNA may potentially bind to and modulate the translation of many different mRNA targets; in addition, one mRNA may contain binding sites and thus be a target for many different miRNAs [34,35]. The high complexity of miRNA-mRNA regulatory networks can make it challenging to generate hypotheses for studies on miRNA expression and regulation. Small RNA sequencing (RNAseq) offers a hypothesis-free approach for researchers to investigate miRNA expression, as this method does not require any sequence specific primers or probes in order to perform miRNA quantification, in contrast to more traditional gene expression methodologies such as microarrays or reverse transcription quantitative real-time PCR (RT-qPCR). However, studies that comprehensively compare and validate miRNA expression results obtained by RNAseq and qPCR are scarce but greatly needed in order to generate valid and reproducible data in miRNA research.

## Materials and methods

**Fig 1:**
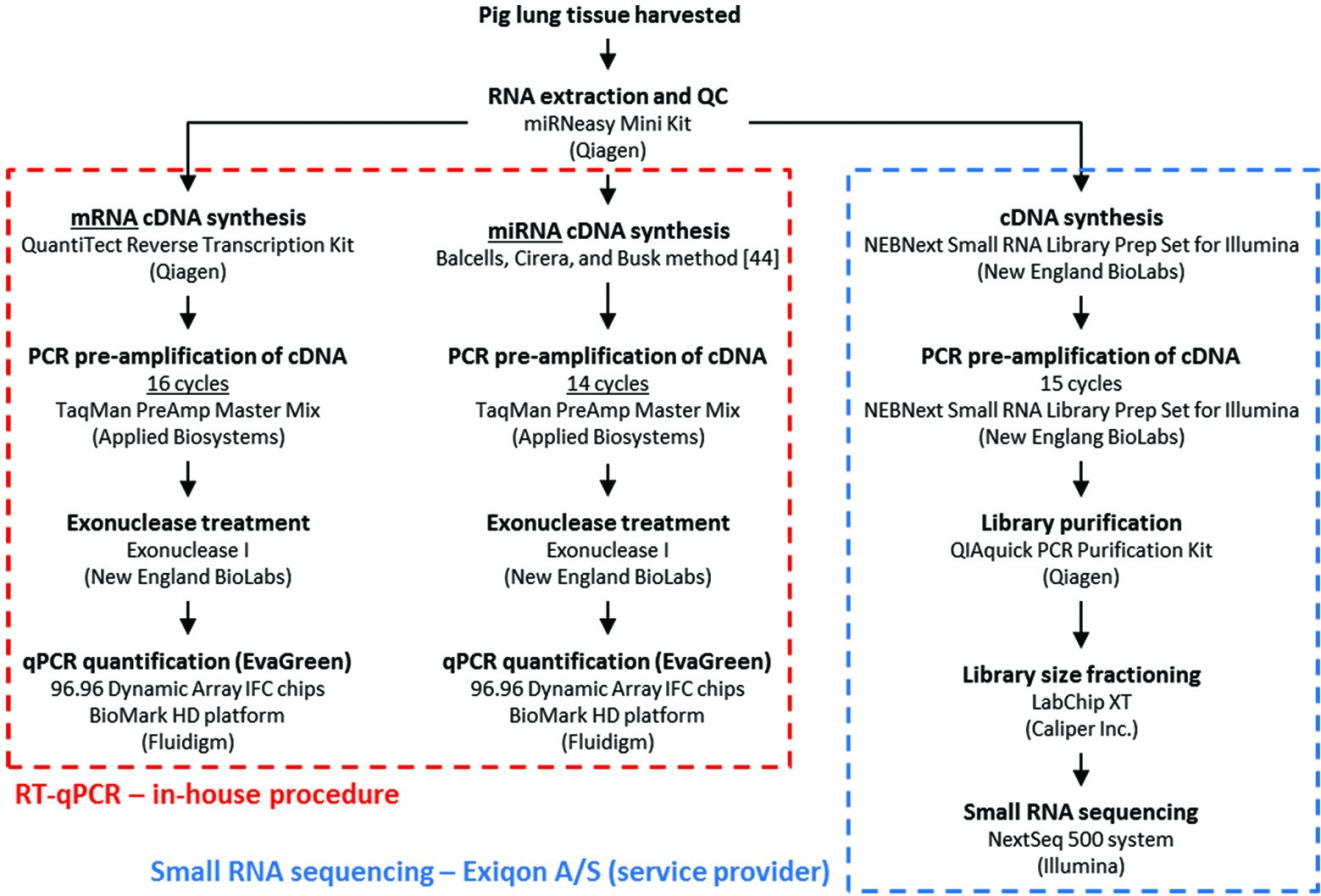
Overview of experimental procedures. RNA extraction and RT-qPCR procedures (boxed in red) were performed in-house, whereas small RNA sequencing (boxed in blue) was performed by an external service provider (Exiqon A/S).

Methods and analyses are described here briefly and summarized in Fig 1; fully detailed descriptions are contained in S1 Appendix.

### Animals and challenge

The challenge experiment will be described briefly, as details have been described previously [8]. All procedures and animal care was carried out in accordance with Good Clinical Practice (VICH GL9, CVMP/VICH/595/98), the Directive 2001/82/EC on the Community code relating to veterinary medicinal products, and German Animal Protection Law. The protocol IDT A 03/2004 was approved by the Landesverwaltungsamt Sachsen-Anhalt, Germany (Reference Number: AZ 42502-3-401 IDT). 25 pigs were included in the present study. 20 pigs were experimentally infected by aerosol exposure (6 l cell culture supernatant containing 10^4.55^ TCID_50_/ml) to the Danish swine IAV strain A/sw/Denmark/12687/03 (H1N2) [36], and 5 unchallenged animals were used as controls; all animals were confirmed seronegative for IAV H1N1, H1N2, and H3N2 subtypes. Clinical signs (body temperature and dyspnea) were recorded during the first 72 h after challenge. Dyspnea was scored as described previously [8]: 0 = breathing unaffected; 1 = increased respiratory frequency and moderate flank movement; 2 = marked pumping breathing and severe flank movement; 3 = labored breathing affecting the entire body, pronounced flank movement and substantial movements of the snout; 4 = severe breathing reflecting substantial lack of oxygen. Infected animals were euthanized at 1 day (n = 6), 3 days (n = 6), and 14 days (n = 8) after challenge. Control animals (n = 5) were euthanized on day 14. Lung tissue samples of 500 mg were collected from the left cranial lobe from regions with no gross lesions, and immediately stabilized in RNA*later* (Qiagen) and stored at -20°C. IAV content in lungs and nasal swabs was determined by RT-qPCR at several time points after challenge as previously described [8].

### RNA extraction

Total RNA was isolated using M-tubes and a gentleMACS Octo Dissociator (Miltenyi Biotec) for tissue homogenization and the miRNeasy Mini Kit (Qiagen) for extraction, according to the manufacturer’s specifications. For each sample, approx. 35 mg lung tissue was homogenized in 1 ml QIAzol Lysis Reagent (Qiagen). The lysate was mixed with 200 μl chloroform and centrifuged at 4°C and 12,000xg for 15 min. The rest of the procedure was carried out at room temperature. 300 μl of the upper aqueous phase was removed and mixed with 450 μl 99.9% ethanol (ratio 1:1.5), and processed in the RNeasy Mini Spin Columns (supplied in kit) as specified by the manufacturer. An on-column DNase treatment was incorporated, using RNase-Free DNase Set (Qiagen); 80 μl DNase solution was pipetted directly onto the column membrane and incubated 15 min. Prior to elution, an additional centrifugation was performed at 12,000xg for 2 min to prevent any carryover of buffers to the elution. RNA was eluted in 50 μl RNase-free water by centrifugation at 8,000xg for 2 min.

RNA purity and concentration was determined using a NanoDrop ND-1000 UV spectrophotometer (Thermo Scientific); mean A260/280 ratio for all RNA samples was 2.1 and mean A260/230 ratio was 2.0, RNA yields ranged from 396 to 1158 ng/μl, mean yield was 753.1 ng/μl. RNA quality was estimated by measuring RNA integrity numbers (RIN) for each extraction, using Agilent RNA 6000 Nano Chips and Agilent RNA 6000 Nano reagents on an Agilent 2100 Bioanalyzer (Agilent Technologies); RIN varied from 5.4 to 8.7, with the mean RIN value being 6.4. The same RNA extractions were applied in both RNAseq and RT-qPCR.

### Small RNA sequencing

All procedures related to library preparation, sequencing, and quality control was carried out by the sequencing provider Exiqon A/S (Vedbaek, Denmark). The RNA was diluted to 250 ng/μl in RNase-free water and a total of 8 μl (2 μg RNA) from each sample was supplied to Exiqon A/S for analysis. For each RNA sample, 1 μg of total RNA was converted into miRNA NGS libraries using the NEBNext Small RNA Library Prep Set for Illumina (New England BioLabs) according to the manufacturer’s instructions. Each individual RNA sample was converted into cDNA and pre-amplified by PCR (protocol and reagents supplied with NEBNext kit). The 3’ PCR primer was associated with a barcode sequence that was unique for each original RNA sample, thus allowing for differentiation of reads from different samples in the subsequent multiplex sequencing procedure of pooled cDNA samples. Libraries were purified using the QIAquick PCR Purification Kit (Qiagen), and the insert efficiency evaluated on a Bioanalyzer 2100 instrument on high sensitivity DNA chips (Agilent Technologies) to confirm a peak at ~140 nt corresponding to the length of adapter-ligated miRNAs. The miRNA cDNA libraries were size fractionated on a LabChip XT (Caliper Inc.) and a band representing adaptors and 15–40 nt insert (i.e. the size range covering miRNAs) excised according to the manufacturer’s instructions. Samples were sequenced on the Illumina NextSeq 500 system, yielding 50 nt single-ended reads of high quality (Q-score above 30). Raw sequencing data were supplied to the authors as compressed FASTQ files.

#### Small RNA sequencing data analysis

**Fig 2:**
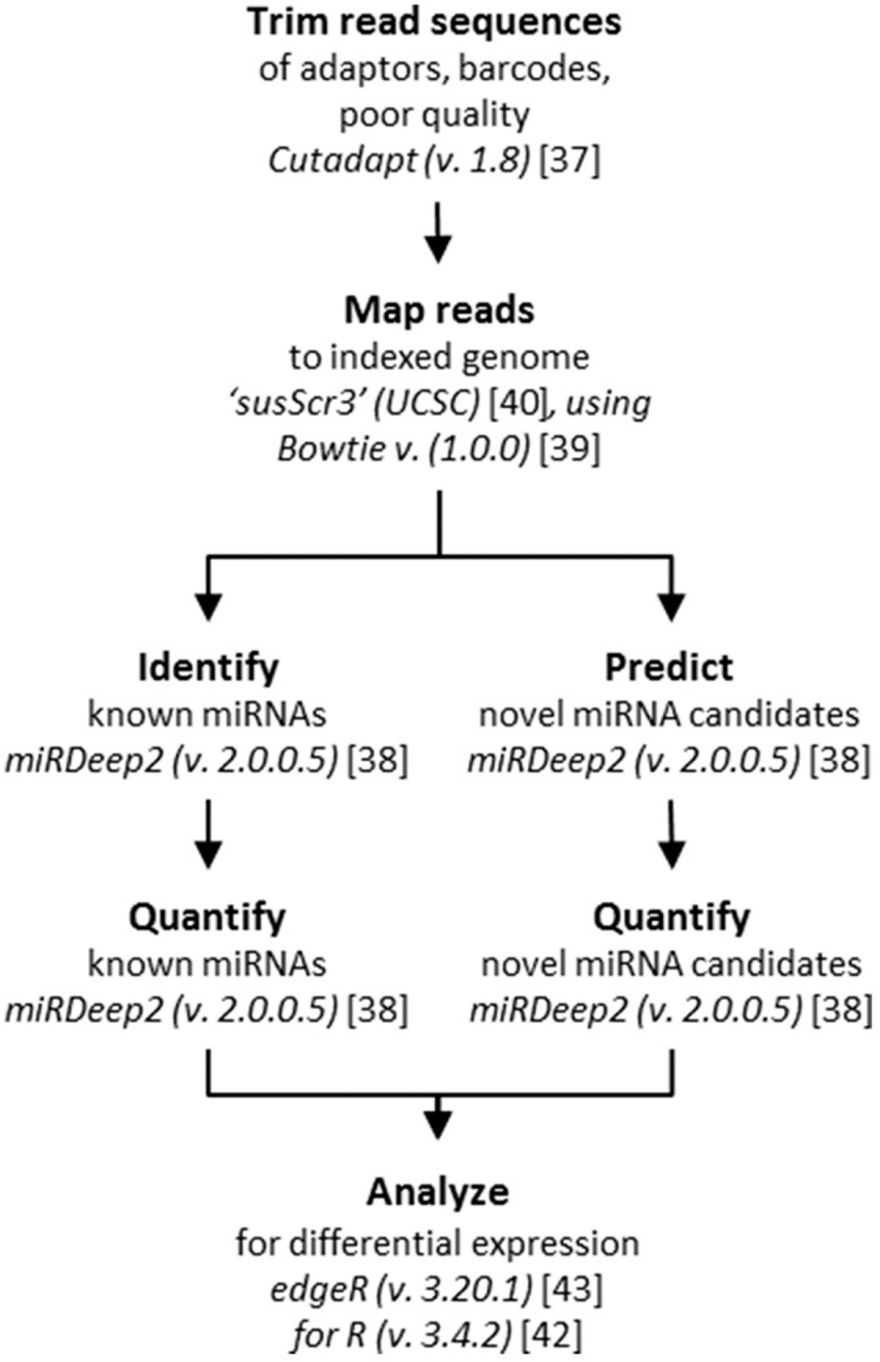
Overview of RNAseq data analysis. Workflow of the bioinformatics tools applied for RNAseq data analysis.

RNAseq data analysis is summarized in Fig 2. Adaptor sequences from the library preparation and sequencing were trimmed from the reads using Cutadapt v. 1.8 [37], retaining only reads with a minimum length of 16 nt. Modules from the miRDeep2 package v. 2.0.0.5 was applied for the remaining sequencing data processing [38], using the mapper module to collapse trimmed reads and Bowtie v. 1.0.0 [39] for the mapping of reads to a pre-built indexed porcine genome from the iGenomes collection from Illumina (‘susScr3’ from UCSC built on the Sscrofa10.2 genome assembly [40], downloaded January 27^th^ 2015 from here: https://support.illumina.com/sequencing/sequencing_software/igenome.html). The miRDeep2 module was supplied with .fa files with trimmed collapsed sequencing reads and the indexed porcine genome (see above), .arf file with reads mapped to the genome, and .fa files with known porcine miRNA sequences and known *Homo sapiens*, *Bos taurus*, and *Mus musculus* miRNA sequences, respectively. The latter was applied for the identification of potential novel (not yet annotated) porcine miRNAs candidates by comparing the obtained read sequences to known mature miRNAs from other species. All miRNA sequences were acquired from miRBase v. 21 [41].

Read counts were obtained from the miRDeep2 output file (.csv), and the remaining analysis for differential expression and statistical analysis was performed in R (v. 3.4.2) [42] using the package edgeR (v. 3.20.1) [43] and applying the general linearized model (glm) approach, testing for significant differential expression between groups using an *F*-test. The Benjamini-Hochberg procedure was applied to control the false discovery rate (FDR) at α = 0.05. miRNA regulation was calculated as log_2_(fold change) (log_2_FC) of expression levels in a post-challenge group relative to the control group. A miRNA was considered to be differentially expressed if it was ≥50% up- or down-regulated, i.e. log_2_FC > 0.585 or log_2_FC < -0.585, and significant in an *F*-test (*p* < 0.05).

### miRNA RT-qPCR

100 ng total RNA obtained from IAV infected porcine lung samples were reverse transcribed into cDNA and pre-amplified prior to high-throughput qPCR using the method described by Balcells, Cirera, and Busk [44], thus carrying out polyadenylation and reverse transcription in the same reaction.–RT (reverse transcriptase replaced with water) and–poly(A) (poly(A) polymerase replaced with water) controls were included. Two cDNA replicates were synthesized from each RNA sample. Residual primers were digested by Exonuclease I (New England BioLabs) treatment. All miRNA primers, including the universal RT primer, were designed as described previously [44] and purchased from Sigma-Aldrich. Whenever a porcine version of a miRNA was and available in miRBase at the time of primer design, this sequence was used (indicated by the prefix ssc). Otherwise, the sequence of the human (hsa) (or mouse, mmu, in the case of novel miRNAs from sequencing data) homolog was used instead. Sequences and qPCR efficiencies for all primers can be found in S1 Table. The majority of miRNAs assayed by RT-qPCR were chosen based on RNAseq results. Additional miRNAs not detected by sequencing were also included in the qPCR analysis, based on our previous work and literature studies. qPCR was carried out in 96.96 Dynamic Array IFC chips using the high-throughput BioMark HD real-time platform (Fluidigm). 96 sample mixes (including a non-template control, using water instead of cDNA) and 96 primer mixes were added to the 96.96 Dynamic Array IFC chip, and a total of 9,216 qPCR reactions were carried out in parallel. Dilution series were prepared from a pool of all samples (except –RT and –poly(A) controls) and included in the qPCR to assess primer efficiency. Melting curve analysis was performed lastly to assess qPCR specificity.

#### miRNA qPCR data analysis

All amplification and melting curves were visually inspected using the Fluidigm Real-Time PCR Analysis software (v. 4.1.3). Primer efficiency was assessed for each assay from the standard curves produced from the included dilution series and subsequently used to adjust the C_q_ values in the individual assays. Primer efficiencies ranged from 92 to 119%. miRNA qPCR data was normalized using the global mean expression method [45] and cDNA replicates were averaged. C_q_ values were converted to relative quantities on the linear scale. miRNA regulation was calculated as log_2_FC of expression levels in a post-challenge group relative to the control group. A miRNA was considered to be differentially expressed if it was ≥50% up- or down-regulated, i.e. log_2_FC > 0.585 or log_2_FC < -0.585, and significant in a *t*-test (*p* < 0.05, log_2_ transformed data), using the T.TEST() function in Microsoft Excel (two-tailed, equal variance).

### RT-qPCR of protein coding genes

For mRNA, the cDNA synthesis was performed with 500 ng RNA using the QuantiTect Reverse Transcription Kit (Qiagen), including DNase treatment with gDNA Wipeout Buffer (Qiagen).–RT controls (reverse transcriptase replaced with water) were included. Two cDNA replicates were synthesized from each RNA sample. All cDNA samples were pre-amplified prior to qPCR using TaqMan PreAmp Master Mix (Applied Biosystems) and a pool of all primers to be included in the subsequent qPCR (at 200 nM each). Highly specific mRNA primers were designed according to specification previously described [46] using Primer3 (http://bioinfo.ut.ee/primer3-0.4.0/). Included in the panel of assayed genes were a number of potential reference genes (‘housekeeping genes’) that would later be evaluated for their suitability for data normalization. Dilution series were prepared from a pool of all samples and included in the qPCR (except the –RT control) to assess primer efficiency. Sequences and qPCR efficiencies for all primers can be found in S1 Table. High-throughput qPCR was carried out on the BioMark HD real-time instrument (Fluidigm) in a setup identical to that described for miRNA (section ‘miRNA RT-qPCR’).

#### mRNA qPCR data analysis

All amplification and melting curves were visually inspected using the Fluidigm Real-Time PCR Analysis software (v. 4.1.3). Primer efficiency was assessed from the standard curves used to adjust the C_q_ values in the individual assays. Primer efficiencies ranged from 95 to 113%. Potential reference genes were evaluated using the algorithms geNorm [47] and NormFinder [48], and β-actin (*ACTB*), β_2_ microglobulin (*B2M*), glyceraldehyde 3-phosphate dehydrogenase (*GAPDH*), peptidylprolyl isomerase A (*PPIA*), 60S ribosomal protein L13A (*RPL13A*), and tyrosine 3-monooxygenase/tryptophan 5-monooxygenase activation protein, zeta polypeptide (*YWHAZ*) were applied for mRNA qPCR data normalization. cDNA replicates were averaged and C_q_ values were converted to relative quantities on the linear scale. mRNA regulation was calculated as log_2_FC of expression levels in a post-challenge group relative to the control group. A protein coding gene was considered to be differentially expressed if it was ≥100% up- or down-regulated, i.e. log_2_FC > 1 or log_2_FC < -1, and significant in a *t*-test (*p* < 0.05, log_2_ transformed data), using the T.TEST() function in Microsoft Excel (two-tailed, equal variance).

### miRNA-mRNA interactions

Using online tools and databases (see below), potential mRNA targets among the assayed protein coding genes were identified for miRNAs found to be differentially expressed after IAV challenge. As very little experimental target validation has been performed for porcine miRNAs, the human miRNA homologs as well as protein coding genes were applied for target identification and prediction. A comparison of porcine and human miRNA sequences applied in miRNA-mRNA interaction analysis can be found in S2 Table, where the seed sequences (nucleotides 2–7) have been highlighted, as these nucleotides are primarily responsible for miRNA targeting specificity. One differentially expressed miRNA was excluded from these analyses, as no human homolog has yet been annotated (ssc-miR-7134-5p, first identified in a previous study of ours [49]). Previously experimentally validated and computationally predicted targets were identified by using each miRNA as input in a ‘single name’ search in the RAIN database v. 1.0 [50], which integrates non-coding RNA interactions and associations with the protein-protein interaction database STRING v. 10.5 [51]. As RAIN due to licensing restrictions does not integrate information from TarBase v. 7.0 [52] or target predictions by microT-CDS v. 5.0 [53] from DIANA Lab, previously experimentally validated and computationally predicted targets were likewise identified from these two sources. For all validated or predicted miRNA-mRNA interactions obtained from RAIN, TarBase, and microT-CDS, Pearson’s correlation coefficient (Pearson’s *r*) was calculated for miRNA and mRNA expression data (qPCR) on day 1 and 3 post challenge. If a negative correlation of *r* < -0.532 (*p* < 0.05) was seen, that particular miRNA-mRNA interaction was regarded as potentially relevant in the regulation of innate immune response in pigs during active IAV infection.

The RAIN database v. 1.0 [50] was likewise applied to identify cellular pathways that could potentially be affected by the differentially expressed miRNAs. Two subsets of differentially expressed miRNAs were applied as input in ‘multiple names’ search in RAIN: 1) miRNAs up-regulated during acute infection on day 1 and/or 3 after challenge and 2) miRNAs down-regulated during acute infection on day 1 and/or 3 after challenge. KEGG Pathway enrichment analysis [54] (integrated in STRING) was performed on the collection of targets identified for each set of the differentially expressed miRNAs.

## Results

### Animals and challenge

Clinical signs of IAV infection were observed in infected animals during the three first days following challenge. Fever peaked on day 1 after challenge with a mean temperature of 41°C, which dropped to 39.9°C on day 3. Dyspnea was likewise most severe on day 1 with a mean score of 2.4, decreasing to 0.8 on day 3. No clinical signs were observed in control animals. As reported previously [8], the challenge strain could be detected by qPCR in lung tissue on day 1 and 3 after challenge and in nasal swabs on day 1, 3, 5, and 7 after challenge. No IAV could be detected on day 14 in nasal swabs or lung tissue [8].

### Small RNA sequencing

Length distribution of reads (after adapter trimming) showed an expected peak around 18–23 nt, corresponding to typical miRNA length. The total number of reads obtained after sequencing ranged from 10.5 to 26.2 million per sample, averaging on 19.5 million. Of these, an average of 14.9 million reads mapped to the porcine genome, with an average of 7.5 million reads mapping to known miRNAs (details for each sample provided in S3 Table). A total of 238 mature annotated porcine miRNAs could be detected in all of the samples (see S4 Table).

61 novel mature miRNA candidate sequences were obtained from the miRDeep2 pipeline and subjected to BLASTN search in the miRBase database (v. 21) [41] to identify miRNA homologs in other species. The best matching human homolog was chosen (highest score, lowest *E*-value), unless a homolog from another species was a better match than the human homolog (higher score, lower *E*-value). Of the 61 candidate sequences, human homologs were identified for 12, mouse homologs for two, and a bovine homolog for one sequence, respectively (Table 1). More details (including candidate hairpin sequences and scores and *E*-values from BLASTN) can be found in S5 Table. These 15 miRNA homologs were subsequently included in qPCR analyses.

**Table 1:** Predicted novel porcine miRNA candidates and their known miRNA homologs.

Novel porcine miRNA candidate	Novel porcine miRNA candidate sequence identified in RNAseq data	Homolog miRNA	Homolog accession number	Homolog miRNA sequence
ssc-miR-novel-1	uucaaguaauucaggauagguu	bta-miR-26b	MIMAT0003531	uucaaguaauucaggauagguu
ssc-miR-novel-2	aaucacuaauuccacugccauc	mmu-miR-34b-3p	MIMAT0000382	aaucacuaacuccacugccauc
ssc-miR-novel-3*	aggcaguguaauuagcugauug**u**	mmu-miR-34b-5p	MIMAT0004581	aggcaguguaauuagcugauug
ssc-miR-novel-4*	uaacacugucugguaaagaug	hsa-miR-141-3p	MIMAT0000432	uaacacugucugguaaagaug**g**
ssc-miR-novel-5	caucuuccagcacaguguugga	hsa-miR-141-5p	MIMAT0004598	caucuuccaguacaguguugga
ssc-miR-novel-6	caucccuugcaugguggaggg	hsa-miR-188-5p	MIMAT0000457	caucccuugcaugguggaggg
ssc-miR-novel-7	uaauacugccugguaaugauga	hsa-miR-200b-3p	MIMAT0000318	uaauacugccugguaaugauga
ssc-miR-novel-8	caucuuacugggcagcauugga	hsa-miR-200b-5p	MIMAT0004571	caucuuacugggcagcauugga
ssc-miR-novel-9	uaauacugccggguaaugaugga	hsa-miR-200c-3p	MIMAT0000617	uaauacugccggguaaugaugga
ssc-miR-novel-10	uuuguucguucggcucgcguga	hsa-miR-375	MIMAT0000728	uuuguucguucggcucgcguga
ssc-miR-novel-11	uggcaguguauuguuagcuggu	hsa-miR-449a	MIMAT0001541	uggcaguguauuguuagcuggu
ssc-miR-novel-12	aggcaguguauuguuagcuggc	hsa-miR-449b-5p	MIMAT0003327	aggcaguguauuguuagcuggc
ssc-miR-novel-13	uagugcaauauugcuuauagggu	hsa-miR-454-3p	MIMAT0003885	uagugcaauauugcuuauagggu
ssc-miR-novel-14	uaauuuuauguauaagcuagu	hsa-miR-590-3p	MIMAT0004801	uaauuuuauguauaagcuagu
ssc-miR-novel-15	uacccauugcauaucggaguug	hsa-miR-660-5p	MIMAT0003338	uacccauugcauaucggaguug

When equally identical homologs were found in several species, the human homolog is shown here. Species specific prefixes are as follows: ssc—*Sus scrofa*, hsa—*Homo sapiens*, bta—*Bos taurus*, and mmu—*Mus musculus*.

* indicates that the novel porcine sequence is not 100% identical to its matching homolog (see underlined bold nucleotides).

**Fig 3:**
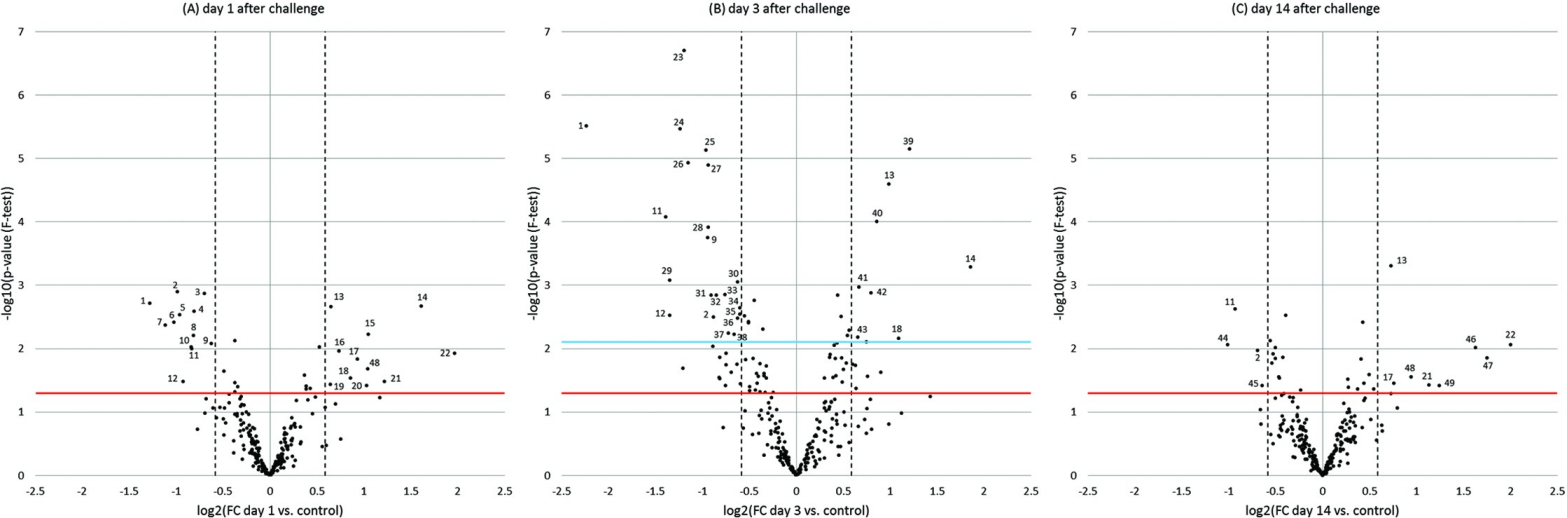
Volcano plots showing miRNA expression changes as obtained by RNAseq. A) day 1 after challenge, B) day 3 after challenge, C) day 14 after challenge. x-axes show the log_2_FC values of post-challenge time points vs. unchallenged controls. 50% up- or down-regulation is denoted by vertical dotted lines (log_2_FC > 0.585, log_2_FC < -0.585). y-axes show the -log_10_ transformed *p*-values obtained from *F*-tests. *p* = 0.05 is denoted by a horizontal red line. A horizontal blue line denotes the *p*-value limit for significant differential expression when controlling for FDR; as shown, only on day 3 after challenge are there any miRNAs that are significantly differentially expressed after this correction. miRNAs that pass the criteria for differential expression are marked with a number denoting their identity; miRNAs that appear in more than one volcano plot are marked with the same number in all plots. 1 ssc-miR-205; 2 ssc-miR-34c; 3 ssc-miR-671-5p; 4 ssc-miR-146a-5p; 5 ssc-miR-2366; 6 ssc-miR-365-5p; 7 ssc-miR-146b; 8 ssc-miR-708-5p; 9 ssc-miR-92b-5p; 10 ssc-miR-708-3p; 11 mmu-miR-34b-3p; 12 mmu-miR-34b-5p; 13 ssc-miR-339-3p; 14 ssc-miR-196b-5p; 15 ssc-miR-193a-3p; 16 ssc-miR-4334-3p; 17 ssc-miR-133b; 18 ssc-miR-217; 19 ssc-miR-331-3p; 20 ssc-miR-216; 21 ssc-miR-187; 22 ssc-miR-144; 23 ssc-miR-296-3p; 24 ssc-miR-92b-3p; 25 ssc-miR-1343; 26 hsa-miR-375; 27 ssc-miR-744; 28 ssc-miR-7134-5p; 29 ssc-miR-190b; 30 ssc-miR-2320-3p; 31 ssc-miR-671-3p; 32 ssc-miR-128; 33 ssc-miR-328; 34 ssc-miR-30c-1-3p; 35 ssc-miR-1296-5p; 36 ssc-miR-149; 37 ssc-miR-183; 38 ssc-miR-129b; 39 ssc-miR-221-5p; 40 ssc-miR-29b; 41 ssc-miR-142-5p; 42 ssc-miR-504; 43 ssc-miR-20a; 44 ssc-miR-1249; 45 ssc-miR-7139-3p; 46 ssc-miR-486; 47 ssc-miR-451; 48 ssc-miR-335; 49 ssc-miR-215.

**Fig 4:**
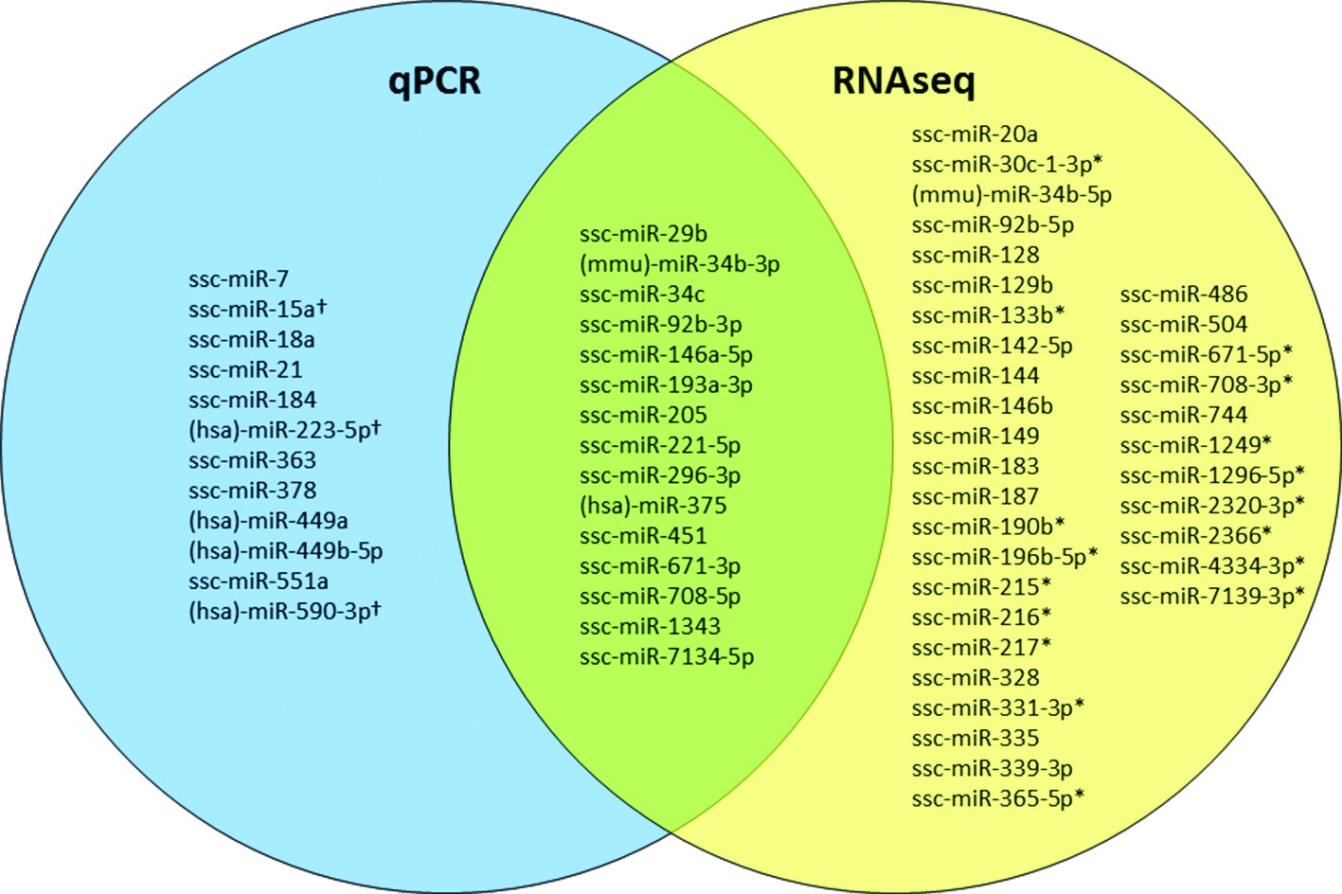
Venn diagram comparing differentially expressed miRNAs identified by and qPCR and RNAseq. Overlap of miRNAs found to be differentially expressed at one or more post-challenge time points by RNAseq (yellow) and qPCR (blue). †miRNAs assayed by qPCR but not detected by sequencing; *miRNAs detected by sequencing, but not assayed by qPCR. When a known porcine (ssc) sequence for a given miRNA was not available in miRBase (v. 21), human (hsa) or mouse (mmu) names are applied in accordance with the homolog that best matched the novel porcine miRNA discovered in the RNAseq data, and these homolog sequences were likewise used for qPCR primer design.

Fold changes of miRNA expression (known and novel candidates) in lung tissue of infected animals relative to the uninfected control animals were calculated. These results are shown in volcano plots in Fig 3, highlighting the number of differentially expressed miRNAs at each of the post-challenge time points, i.e. statistically significant up- or down-regulation. Log_2_FC of all detected miRNAs are summarized in S6 Table. A total of 49 miRNAs were found to be differentially expressed at one or more time points (*p* < 0.05) (Fig 4).

### Comparison of RNAseq and qPCR results

**Fig 5:**
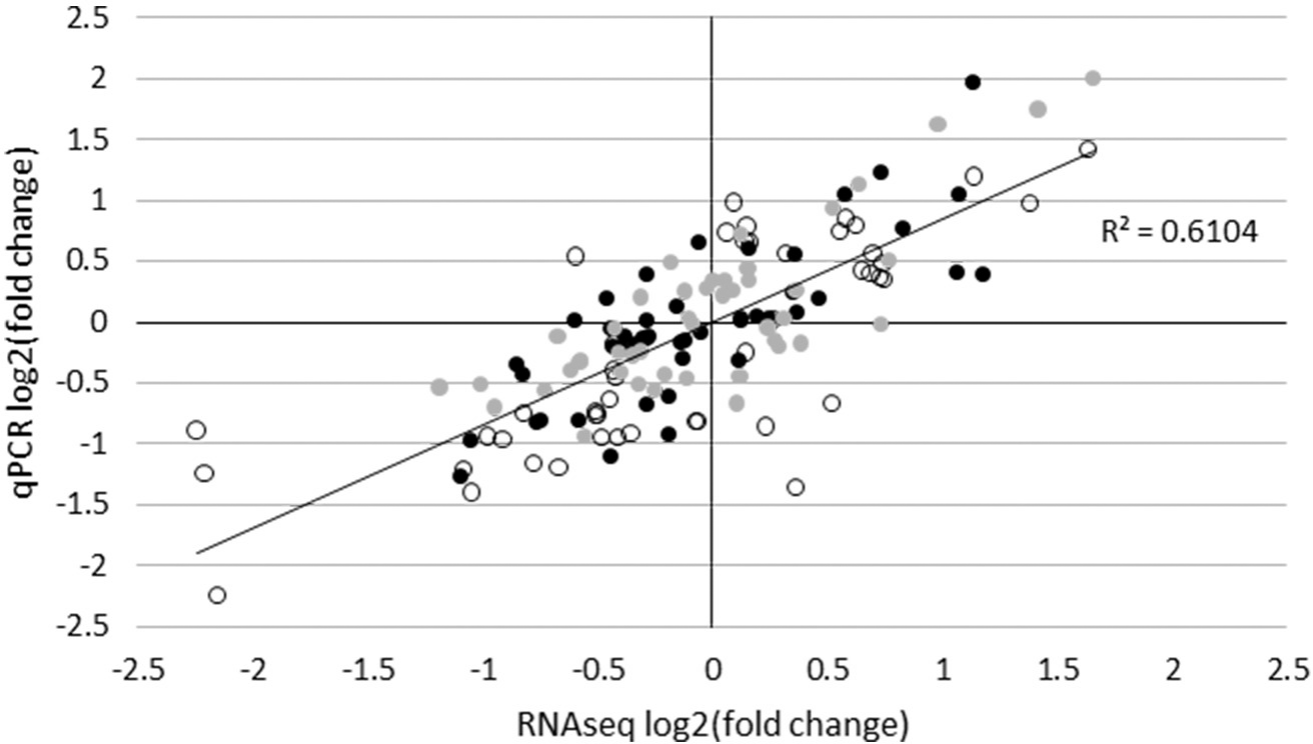
Comparison of miRNA log_2_FC as measured by RNAseq and qPCR. Log_2_FC (challenged group vs. control group) obtained by qPCR is plotted against the corresponding log_2_FC obtained by RNAseq. Each point represents log_2_FC of one miRNA on day 1 (black), day 3 (white), or day 14 (grey) after challenge.

qPCR expression data was obtained for a total of 80 miRNA (see S7 Table); 27 of these met the criteria for being differentially expressed (log_2_FC > 0.585 or log_2_FC < -0.585, *p* < 0.05 (*t*-test)) (Table 2). Generally, good agreement was found between RNAseq and qPCR results. For those miRNAs found to be differentially expressed at one or more time points after challenge by at least one of the methods, a significant positive sample-wise correlation (Pearson’s *r*, *p* < 0.05) of normalized RNAseq and qPCR expression values was seen for 74% of the miRNAs. Expression patterns for miRNAs found to be differentially expressed at one or more time points after challenge obtained with the two methods are compared in Fig 5. Linear regression showed an R^2^ of 0.6104, and a highly significant positive correlation (Pearson’s *r*) (*r* = 0.78, *p* < 0.01).

Results from both methods concur that day 3 is the time point where there is the highest number of differentially expressed miRNAs (Table 2). qPCR analysis on day 3 showed 19 miRNAs to be differentially expressed relative to the control group; only roughly half as many were differentially expressed on day 1 and 14. Only two miRNAs were significantly changed throughout the whole experiment: ssc-miR-34c and ssc-miR-92b-3p. Both were down-regulated at very similar levels at all post-challenge time points.

**Table 2:** Differential expression of miRNAs measured by qPCR in pig lungs after IAV challenge relative to unchallenged controls.

	Day 1 after challenge relative to control group		Day 3 after challenge relative to control group		Day 14 after challenge relative to control group	
miRNA	log_2_FC (lower 95% CI; upper 95% CI)	*p* (*t*-test)	log_2_FC (lower 95% CI; upper 95% CI)	*p* (*t*-test)	log_2_FC (lower 95% CI; upper 95% CI)	*p* (*t*-test)
ssc-miR-7	**1.2 (0.79; 1.5)**	**0.0019**	0.70 (0.15; 1.1)	0.065	-0.43 (-0.67; -0.22)	0.13
ssc-miR-15a†	0.41 (0.25; 0.56)	0.0070	**0.63 (0.53; 0.73)**	**8.9E-05**	0.23 (0.049; 0.39)	0.10
ssc-miR-18a	0.12 (-0.16; 0.37)	0.57	**0.65 (0.45; 0.81)**	**0.0033**	0.36 (0.18; 0.53)	0.043
ssc-miR-21	-0.12 (-0.39; 0.11)	0.50	**0.75 (0.65; 0.84)**	**0.00016**	0.27 (0.085; 0.44)	0.096
ssc-miR-29b	0.37 (0.17; 0.54)	0.020	**0.58 (0.38; 0.76)**	**0.0014**	-0.12 (-0.30; 0.038)	0.28
mmu-miR-34b-3p	-0.76 (-1.5; -0.28)	0.058	**-1.0 (-1.3; -0.85)**	**0.0019**	-0.56 (-0.93; -0.26)	0.079
ssc-miR-34c	**-1.0 (-1.5; -0.67)**	**0.0026**	**-2.2 (-3.3; -1.6)**	**0.00026**	**-0.95 (-1.3; -0.66)**	**0.0029**
ssc-miR-92b-3p	**-0.85 (-1.2; -0.57)**	**0.0045**	**-2.2 (-2.6; -1.9)**	**3.4E-06**	**-1.2 (-1.4; -0.98)**	**9.4E-05**
ssc-miR-146a-5p	**-0.58 (-0.91; -0.30)**	**0.034**	-0.43 (-0.86; -0.098)	0.10	-0.087 (-0.35; 0.13)	0.65
ssc-miR-184	-0.60 (-1.9; 0.072)	0.27	**-1.1 (-1.5; -0.78)**	**0.047**	-0.25 (-0.70; 0.094)	0.74
ssc-miR-193a-3p	**0.58 (0.36; 0.78)**	**0.0028**	**0.73 (0.43; 0.97)**	**0.0017**	0.16 (-0.10; 0.38)	0.46
ssc-miR-205	**-1.1 (-1.8; -0.64)**	**0.028**	**-2.2 (-2.7; -1.8)**	**0.00021**	0.088 (-0.41; 0.46)	0.84
ssc-miR-221-5p	**1.1 (0.56; 1.5)**	**0.0066**	**1.1 (0.15; 1.7)**	**0.040**	-0.31 (-1.2; 0.22)	0.27
hsa-miR-223-5p†	0.57 (0.32; 0.79)	0.023	**1.1 (0.86; 1.4)**	**0.00042**	0.17 (-0.068; 0.37)	0.40
ssc-miR-296-3p	-0.12 (-0.38; 0.10)	0.58	**-0.67 (-0.96; -0.43)**	**0.016**	**-0.57 (-0.82; -0.36)**	**0.015**
ssc-miR-363	0.20 (-0.057; 0.42)	0.40	**0.69 (0.44; 0.90)**	**0.011**	-0.10 (-0.33; 0.095)	0.76
hsa-miR-375	**-0.82 (-1.1; -0.61)**	**0.00031**	**-0.78 (-1.2; -0.43)**	**0.0053**	-0.11 (-0.26; 0.023)	0.30
ssc-miR-378	-0.28 (-0.40; -0.17)	0.18	-0.42 (-0.47; -0.38)	0.042	**-0.62 (-0.91; -0.37)**	**0.018**
hsa-miR-449a	-0.28 (-0.78; 0.086)	0.25	-0.50 (-1.1; -0.059)	0.10	**0.77 (0.47; 1.0)**	**0.0060**
hsa-miR-449b-5p	-0.38 (-0.90; 0.0040)	0.18	-0.59 (-1.2; -0.16)	0.076	**0.73 (0.48; 0.95)**	**0.0058**
ssc-miR-451	0.84 (-0.71; 1.6)	0.20	1.4 (-1.2; 2.3)	0.080	**1.4 (0.37; 2.0)**	**0.045**
ssc-miR-551a	-0.15 (-0.40; 0.059)	0.35	**-0.82 (-1.1; -0.57)**	**0.0035**	**-1.0 (-1.3; -0.75)**	**0.00022**
hsa-miR-590-3p†	0.36 (0.049; 0.61)	0.14	**0.74 (0.23; 1.1)**	**0.027**	-0.026 (-0.35; 0.24)	0.86
ssc-miR-671-3p	-0.43 (-0.69; -0.21)	0.021	-0.36 (-0.66; -0.11)	0.057	**-0.58 (-0.69; -0.48)**	**6.7E-05**
ssc-miR-708-5p	**-0.74 (-0.90: -0.60)**	**0.0022**	-0.43 (-0.88; -0.080)	0.14	**-0.67 (-1.0; -0.41)**	**0.014**
ssc-miR-1343	-0.40 (-0.69; -0.15)	0.073	**-0.91 (-1.2; -0.66)**	**0.0016**	-0.21 (-0.38; -0.058)	0.23
ssc-miR-7134-5p	-0.46 (-0.82; -0.17)	0.040	**-0.98 (-1.2; -0.79)**	**0.00013**	**-0.73 (-0.91; -0.57)**	**0.00035**

Log_2_FC of miRNA expression on day 1, 3, and 14 after IAV challenge relative to unchallenged controls. Lower and upper limits for 95% confidence interval are given in parentheses. Data in bold text are those that meet the criteria for differential expression (see text). *p*-values were obtained from *t*-test between the control group and a post-challenge group. †miRNAs that were not identified in RNAseq data.

### qPCR of protein coding genes

Analysis of differential expression of protein coding genes was focused primarily on factors of the antiviral innate immune system and apoptosis, thus gathering a thorough characterization of the processes that are engaged to combat the IAV infection locally in the lung of our pig model. A total of 49 genes were identified by RT-qPCR as being differentially expressed (log_2_FC > 1 or log_2_FC < -1, *p* < 0.05 (*t*-test)) in the lung at one or more time points after infection (Table 3, full list in S8 Table).

A dramatic interferon response dominated on day 1 after challenge, demonstrated by a log_2_FC of 8.7 and 6.3 for *IL28B* (IFN-λ3) and *IFNB1* (IFN-β), respectively (Table 3). The most strongly up-regulated PRRs were the two cytoplasmic RLRs RIG-I (*DDX58*) and MDA5 (*IFIH1*) (log_2_FC 6.1 and 3.5 on day 1 after challenge, respectively) (S8 Table). Other up-regulated PRRs included TLRs 3 and 7 (log_2_FC 2.5 and 1.8 on day 1 after challenge, respectively) (S8 Table). These four PRRs all remained significantly up-regulated on day 3 after challenge. Several cytokines with both pro- and anti-inflammatory functions were also up-regulated on day 1 and (partly) day 3 after challenge, including *IL1B* (IL-1β), *IL6* (IL-6), *IL1RN* (IL-1RA), and *IL10* (IL-10) (S8 Table). The only cytokine to demonstrate down-regulation in response to IAV infection was the pro-inflammatory *IL18* (IL-18). A strong chemokine response, especially by *CCL2* (CCL2) and *CXCL10* (CXCL10), was also detected on day 1 after challenge (S8 Table). Additionally, the importance of apoptosis in the response to IAV infection was clearly demonstrated by the early up-regulation of e.g. genes related to the Jak/STAT signaling pathway (*STAT1*, *STAT2*, *JAK2*, *IRF2*, *IRF7*, *SOCS1*), as well as caspases 1 and 3 (*CASP1*, *CASP3*), Bcl-2 (*BLC2*), Mcl-1 (*MCL1*), and protein kinase R (*EIF2AK2*) (Table 3).

**Table 3:** Differential expression of protein coding genes in pig lungs after IAV infection relative to unchallenged controls.

		Day 1 after challenge relative to control group		Day 3 after challenge relative to control group		Day 14 after challenge relative to control group	
Gene product	Gene	log_2_(FC) (lower 95% CI; upper 95% CI)	*p* (*t*-test)	log_2_(FC) (lower 95% CI; upper 95% CI)	*p* (*t*-test)	log_2_(FC) (lower 95% CI; upper 95% CI)	*p* (*t*-test)
Interferon and interferon stimulated genes							
IFN-λ3 (interferon-λ3)	*IL28B*	**8.7 (7.8; 9.2)**	**2.2E-08**	**6.1 (3.3; 7.0)**	**0.0014**	1.2 (0.33; 1.7)	0.098
IFN-β (interferon-β)	*IFNB1*	**6.3 (5.3; 6.9)**	**1.5E-06**	**4.6 (3.2; 5.3)**	**0.00057**	0.71 (-0.15; 1.2)	0.43
ISG15 (interferon-stimulated gene 15)	*ISG15*	**6.3 (5.9; 6.7)**	**6.3E-07**	**4.0 (2.6; 4.7)**	**0.012**	1.1 (0.0; 2.6)	0.50
IFITM1 (interferon-induced transmembrane protein 1)	*IFITM1*	**3.6 (3.2; 3.9)**	**7.4E-07**	**2.0 (1.2; 2.5)**	**0.0057**	0.25 (-2.7; 1.1)	0.74
IFITM3 (interferon-induced transmembrane protein 3)	*IFITM3*	**3.3 (2.7; 3.7)**	**0.000010**	**2.1 (1.3; 2.6)**	**0.0025**	0.23 (0.0; 1.2)	0.59
IRF2 (interferon regulatory factor 2)	*IRF2*	**1.8 (1.4; 2.1)**	**0.000060**	0.59 (0.013; 1.0)	0.086	-0.094 (-0.21; 0.012)	0.41
IRF7 (interferon regulatory factor 7)	*IRF7*	**4.7 (4.4; 5.0)**	**1.5E-07**	**3.0 (2.0; 3.6)**	**0.0064**	0.22 (-5.0; 1.2)	0.63
SOCS1 (suppressor of cytokine signalling 1)	*SOCS1*	**3.0 (2.4; 3.4)**	**0.000027**	**1.8 (0.91; 2.3)**	**0.0063**	-0.15 (-0.32; 0.013)	0.61
Mx1 (interferon-induced GTP-binding protein Mx1)	*MX1*	**5.5 (5.1; 5.8)**	**1.6E-07**	**3.4 (2.2; 4.1)**	**0.012**	0.67 (0.0; 1.9)	0.54
OASL (59 kDa 2'-5'-oligoadenylate synthetase-like protein)	*OASL*	**5.3 (4.9; 5.5)**	**2.5E-07**	**4.3 (3.2; 4.9)**	**0.018**	0.47 (0.0; 1.5)	0.73
OAS1 (2'-5'-oligoadenylate synthetase 1)	*OAS1*	**3.5 (3.2; 3.8)**	**3.8E-06**	**1.6 (0.90; 2.1)**	**0.022**	-0.74 (-2.9; 0.091)	0.09
PKR (protein kinase R)	*EIF2AK2*	**3.3 (3.0; 3.6)**	**1.8E-06**	**1.9 (1.1; 2.4)**	**0.015**	-0.16 (-1.3; 0.48)	0.52
RNase L (ribonuclease L)	*RNASEL*	**3.4 (3.0; 3.8)**	**9.6E-06**	1.4 (0.23; 2.0)	0.074	-0.24 (-0.76; 0.14)	0.62
STAT1 (signal transducer and activator of transcription 1)	*STAT1*	**3.4 (3.0; 3.7)**	**1.7E-07**	**2.1 (1.3; 2.5)**	**0.00020**	0.45 (0.10; 0.73)	0.15
STAT2 (signal transducer and activator of transcription 2)	*STAT2*	**2.7 (2.3; 3.0)**	**3.2E-06**	0.94 (0.17; 1.4)	0.11	-0.28 (-0.92; 0.17)	0.20
JAK2 (Janus kinase 2)	*JAK2*	**2.0 (1.6; 2.3)**	**0.0000097**	**1.2 (0.92; 1.4)**	**0.000016**	0.32 (0.13; 0.48)	0.047
Apoptosis							
CASP1 (caspase-1)	*CASP1*	**1.7 (1.3; 2.1)**	**0.0025**	0.69 (-0.075; 1.2)	0.26	-0.45 (-1.1; 0.0071)	0.39
CASP3 (caspase-3)	*CASP3*	**1.8 (1.4; 2.1)**	**0.00014**	**1.1 (0.90; 1.3)**	**0.000046**	0.42 (0.059; 0.70)	0.18
Bcl-2 (B-cell lymphoma 2)	*BCL2*	**2.3 (1.8; 2.7)**	**0.000061**	0.90 (0.45; 1.2)	0.012	0.31 (-0.19; 0.68)	0.45
Mcl-1 (induced myeloid leukemia cell differentiation protein Mcl-1)	*MCL1*	**1.5 (1.1; 1.9)**	**0.00015**	0.69 (0.37; 0.95)	0.0032	0.25 (0.63; 0.41)	0.079
FasR (FAS receptor)	*FAS*	**2.1 (1.4; 2.5)**	**0.00028**	0.93 (0.43; 1.3)	0.018	0.23 (-0.049; 0.47)	0.34
FasL (FAS ligand)	*FASLG*	**1.8 (1.5; 2.0)**	**0.00018**	0.82 (0.10; 1.3)	0.062	-0.16 (-0.45; 0.085)	0.79
GZMB (granzyme B)	*GZMB*	**1.7 (1.5; 1.9)**	**0.00062**	**1.0 (0.64; 1.3)**	**0.017**	-0.12 (-0.30; 0.043)	0.82
Acute phase proteins							
SAA (serum amyloid A)	*SAA*	0.90 (0.43; 1.3)	0.061	-0.12 (-1.0; 0.44)	0.80	**-2.6 (-3.2; -2.1)**	**0.00011**
PAI-1 (plasminogen activator inhibitor-1)	*SERPINE1*	**2.5 (1.9; 3.0)**	**0.000038**	**2.2 (0.70; 2.9)**	**0.0063**	-0.016 (-0.80; 0.49)	0.60
TF (transferrin)	*TF*	-0.90 (-1.3; -0.59)	0.014	**-1.4 (-2.3; -0.85)**	**0.013**	-0.83 (-1.3; -0.45)	0.018

Log_2_FC of mRNA expression on day 1, 3, and 14 after IAV challenge relative to unchallenged controls. Lower and upper limits for 95% confidence interval are given in parentheses. Data in bold text are those that meet the criteria for differential expression (see text). *p*-values were obtained from *t*-test between the control group and a post-challenge group.

Only one of the assayed protein coding genes was regulated on day 14 after challenge: the positive acute-phase protein (APP) serum amyloid A (*SAA*) showed a log_2_FC of -2.6 at this time point (Table 3). *SAA* was not differentially expressed at earlier time points; however, transferrin (*TF*) and PAI-1 (*SERPINE1*) were regulated on day 3 after challenge in accordance with their roles as negative and positive APPs (log_2_FC -1.4 and 2.2, respectively) (Table 3).

### Target identification of differentially expressed miRNAs

**Fig 6:**
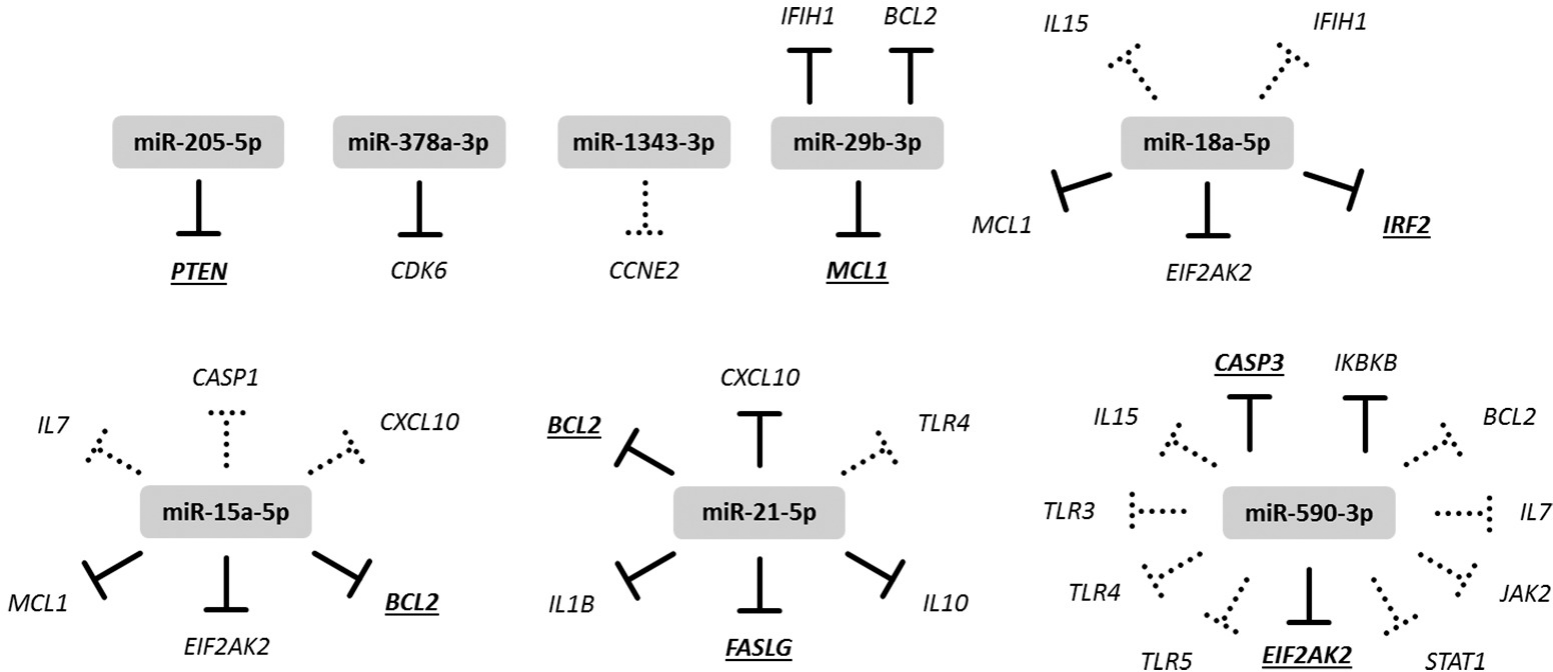
Potential miRNA-mRNA interactions. Expression of eight of the assayed miRNAs showed significant negative correlation with expression of protein coding genes that were identified (using RAIN v1.0 [50], TarBase v. 7.0 [52], and/or microT-CDS [53]) to be experimentally validated targets of the miRNA (solid lines), computationally predicted targets of the miRNA (dotted lines), or both (solid lines, underlined gene names).

Previously experimentally validated or computationally predicted targets (obtained with RAIN, TarBase, and microT-CDS) for the differentially expressed miRNAs among the protein coding genes assayed in the present study was identified using the RAIN database [50], TarBase v. 7.0 [52], and microT-CDS v. 5.0 [53]. For a number of these miRNA-mRNA interactions, a significant negative correlation (*r* < - 0.532, *p* < 0.05) was seen for the expression during active viral infection on day 1 and 3 post challenge. These interactions are summarized in Fig 6. Potential functional implications of miRNA regulation was assessed by KEGG Pathway enrichment analysis of genes identified with RAIN to be validated or predicted targets of the subsets of miRNAs found to be up- or down-regulated during acute infection on day 1 and 3 after challenge. Substantial overlap of enriched pathways was seen for those two sets of target genes, including ‘Influenza A’ (ID 5164), ‘Apoptosis’ (ID 4210), ‘Jak-STAT signaling’ (ID 4630), ‘NF-κB signaling’ (ID 4064), ‘Cytokine-cytokine receptor interaction’ (ID 4060), ‘Chemokine signaling’ (ID 4062), and ‘Endocytosis’ (4144). Among the enriched pathways of relevance to IAV infection, the following were found to be enriched only in one of sets of target genes: ‘B cell receptor signaling’ (ID 4662), ‘Natural killer cell mediated cytotoxicity’ (ID 4650), and ‘Antigen processing and presentation’ (ID 4612) were enriched only in targets of up-regulated miRNAs on day 1 and 3 after challenge, and ‘Toll-like receptor signaling’ (4620), ‘NOD-like receptor signaling’ (ID 4621), and ‘RIG-I-like receptor signaling’ (ID 4622) were enriched only in targets of down-regulated miRNAs on day 1 and 3 after challenge. The miRNA-target interaction networks that form the basis for the KEGG Pathway enrichment analysis can be viewed in S1 and S2 Figs.

## Discussion

### Temporal dynamics of the antiviral response

Transcriptional analysis showed several known miRNAs and novel miRNA candidates to be differentially expressed in the lungs of pigs experimentally infected with H1N2 swine IAV relative to lung tissue from healthy controls. Differentially expressed miRNAs were identified at all examined post-challenge time points (day 1, 3, and 14 after challenge). To the best of our knowledge, this is the first study to present global characterization of miRNA expression in pig lungs after IAV infection. However, several of the miRNAs we found to be differentially expressed have previously been reported to display similar regulation in mouse lung tissue during acute infection after challenge with seasonal H1N1 [26,55] or pandemic (2009) H1N1 [56]. These include ssc-miR-7 [56], ssc-miR-15a [26], ssc-miR-18a [26,56], ssc-miR-21 [55,56], mmu-miR-34b-3p [26], ssc-miR-34c [26], ssc-miR-92b-3p [26], ssc-miR-193a-3p [55], hsa-miR-449a [26], and ssc-miR-671-3p [26]. Three of the up-regulated miRNAs (ssc-miR-7, ssc-miR-221-5p, and ssc-miR-451) likewise overlapped with miRNAs we have previously found to be up-regulated in necrotic lung tissue compared to visually unaffected lung tissue of pigs experimentally infected with the Gram-negative bacterium *Actinobacillus pleuropneumoniae* [49], suggesting a potential common function of these during both viral and Gram-negative bacterial pulmonary infection in pigs.

The majority of differential miRNA expression was found to occur at day 3 after challenge; this is in contrast to our previous findings of miRNA expression in circulating leukocytes of the same animals, where the number of differentially expressed miRNAs peaked after viral clearance, 14 days after challenge [57]. The role of miRNAs expressed in the lung may thus be more focused on modulating transcripts related to the active viral infection. However, in both infected lung tissue and circulating leukocytes the lowest number of differentially expressed miRNAs was seen on day 1 after challenge, whereas the number of differentially expressed protein coding genes related to the antiviral immune response was at its highest at this time point. The miRNA response in the porcine lung may thus be a secondary response with the aim of containing and balancing the rapid inflammatory response upon IAV infection, which if left unchecked may cause massive tissue damage [58].

In terms of clinical symptoms, IAV infection is a short-lived disease in otherwise healthy individuals. However, we here demonstrated in a relevant porcine model that even on day 14 after challenge (when the virus had been cleared), remodeling of the miRNA expression of the lung tissue persisted. Similar results of miRNA regulation in the lungs of mice at late time points after IAV (H1N1) infection were recently published [59]. These authors demonstrated significantly altered expression of miRNAs at 21 days after IAV challenge, including up-regulation of miR-449a which is in agreement with our results 14 days after challenge in the pig lung. Recuperation from IAV infection thus appears to be a protracted process involving miRNAs both locally at the infection site as well as systemically. Such lingering remodeling of the miRNA landscape after viral infection may have important consequences for lung homeostasis and susceptibility to secondary infections. Additional evidence of long term effects of IAV infection locally in the pig lung is seen in the down-regulation of *SAA* 14 days after challenge. This peculiar finding confirms results we have previously published [8]. This conventionally positive APP showed no significant pulmonary regulation during the acute phase of the disease in the lung of IAV infected pigs. However, several reports confirm elevated levels of SAA in serum from pigs within 2–3 days after IAV (H1N1, H1N2, H3N2) infection [60–62]. Thus, there appear to be a role for hepatically produced SAA during the acute phase of IAV infection in pigs, rather than locally produced pulmonary SAA. SAA has been reported to have chemotactic and pro-inflammatory effects [63]; down-regulation of *SAA* during recuperation may thus be a means to restore lung homeostasis after IAV infection.

### Pulmonary type III interferon in pigs

Despite the important role for type III interferon in IAV infection, the pulmonary IFN-λ response remains uncharacterized in the pig. To the best of our knowledge, the present study is the first to report the regulation of IFN-λ in pigs in response to IAV infection. Type III interferons (IFN-λ1, IFN-λ2, IFN-λ3, and IFN-λ4) are the most recently described members of the interferon family [64]. Like their type I interferon (IFN-α and IFN-β) counterparts, they have been shown to be crucial inducers of the antiviral response upon IAV infection in mice and *in vitro* systems [5]. However, in contrast to the type I interferon receptor, which is expressed on the surface of a wide variety of cells, the type III interferon receptor is considered to be largely restricted to epithelial cell surfaces [64,65]. Lung-infiltrating neutrophils in mice have also been demonstrated to respond to type III interferon [66]. Nevertheless, type III and type I interferon signaling through their respective receptors do activate the same ISGs. Mouse experiments have demonstrated IFN-λ to be important for restriction of viral replication and to be the earliest type of interferons produced in response to IAV infection [66,67]. This antiviral function is mediated via ISGs such as Mx1, PKR, and ISG15 [68], which were all found to be up-regulated in the present study.

Fang *et al*. showed that protein levels of IFN-λ1 were elevated in serum in an influenza patient cohort compared to healthy controls. They furthermore demonstrated that hsa-miR-29 (especially hsa-miR-29b-3p) indirectly mediate *IFNL1* up-regulation by controlling DNA methyltransferase (*DNMT3A* and *DNMT3B*) activity in human pulmonary epithelial cells (A549) and a subsequent IRF3/IRF7 dependent transcription of *IFNL1* via COX2 (*PTGS2*), a potent inflammatory protein in IAV infection [69]. Our results mirror these findings in human patients and cells: IFN-λ (*IL28B*) was >400-fold up-regulated (log_2_FC 8.7) in the porcine lung 1 day after IAV challenge, and remained up-regulated on day 3. *IRF7* was also highly up-regulated at these time points. Finally, we also found ssc-miR-29b to be modestly up-regulated on day 1 and 3 after challenge, suggesting that ssc-miR-29b mediated up-regulation of *IL28B* via COX2 and IRF7 may also contribute to the antiviral response in IAV infection in our pig model.

The prominent up-regulation of type III interferon gene expression compared to type I interferon suggested that the antiviral interferon response may be somewhat tailored to epithelial cells, as these are the cells that primarily express the type III interferon receptor. This suggests a preferred targeting of cells that actually contain the virus, as respiratory epithelial cells are the main site for IAV replication *in vivo* [10]. This tailored approach could contribute to lowered tissue damage and disease severity than what might be expected from a dominating type I interferon response as type I receptors are present on a wider range of cells. Corroborating results were recently published by Galani *et al*., who found that pulmonary type I interferon mediated a stronger pro-inflammatory response and increased immunopathology compared to type III interferons in H1N1 IAV infected mice [66]. Here, in our pig model inducing relatively mild IAV disease, we accordingly found the type III interferon to be the dominating response. It can be speculated that the severity of IAV infection relates to the balance between the type I and III interferon responses, with the more severe clinical and immunopathological manifestations being associated with at higher type I interferon response.

### miRNA modulation of the antiviral response

In addition to the abovementioned possible involvement of ssc-miR-29b in the IFN-λ3 response, our results likewise suggested miRNA-mediated modulation of other branches of the innate response to IAV infection in the pig. A number of the assayed protein coding genes were found to be targets (either previously experimentally validated or computationally predicted) of the miRNAs found to be differentially expressed after IAV infection in pigs. Our results supported that some of these interactions are potentially important for the porcine pulmonary response to IAV infection, given that the expression of eight of these miRNAs and their target mRNAs was significantly and negatively correlated during active infection (day 1 and 3 after challenge only). Prominent among the miRNA targeted genes were antiviral PRRs (*IFIH1* and *TLR3*) and genes related to apoptosis (*BCL2*, *MCL1*, *CASP1*, *CASP3*, *FASLG*, and *EIF2AK2*). It is well established that apoptosis occurs in the host during IAV infection, both as a host response to limit viral replication and spread, but also directly induced by viral proteins for the benefit of the virus [10,70,71]. Common to all five miRNAs found to correlate negatively with apoptosis-related gene expression (ssc-miR-15a, ssc-miR-18a, ssc-miR-21, ssc-miR-29b, and hsa-miR-590-3p) is that they were significantly up-regulated on day 3 after challenge, and not regulated at other time points. This concurs with the expression of the apoptosis-related genes, all down-regulated at day 3 compared to their initial up-regulation on day 1. The same pattern holds true for the expression *IFIH1* and *TLR3* and the miRNAs that target them (ssc-miR-29b, ssc-miR-18a, and hsa-miR-590-3p). This negative correlation between miRNA expression and their target transcripts thus suggest the specific involvement of ssc-miR-15a, ssc-miR-18a, ssc-miR-21, ssc-miR-29b, and hsa-miR-590-3p in the modulation of important signaling cascades of apoptosis and viral recognition. Given the likely suppressive effect of these miRNAs on their target genes’ protein output, the miRNA-mediated down-regulation of *IFIH1* and *TLR3* may be way of limiting the pro-inflammatory effects of viral PRR signaling upon IAV infection in order to limit immunopathology. Concordantly, the pro-inflammatory factors *IL1B* and *CXCL10* are likewise among the miRNA-targeted genes showing significant negative correlation with one or more miRNAs and a strong up-regulation on day 1 after challenge but not on day 3.

Gene targets for the differentially expressed miRNAs were identified using online databases and prediction tools, and subjected to KEGG Pathway enrichment analysis. A large overlap of enriched pathways relevant to IAV infection was seen for genes targeted by either up- or down-regulated miRNAs on day 1 and 3 after challenge, including ‘Influenza A’, ‘Apoptosis’, ‘Endocytosis’, ‘Jak-STAT signaling’, ‘NF-κB signaling’, ‘Cytokine-cytokine receptor interaction’, and ‘Chemokine signaling’. This coinciding up- and down-regulation of miRNAs that target such central pathways activated during IAV infection exemplifies the role of miRNAs in balancing and fine-tuning the antiviral innate immune response. Interestingly, we found the pathways ‘Toll-like receptor signaling’, ‘NOD-like receptor signaling’, and ‘RIG-I-like receptor signaling’ to be enriched only in targets of miRNAs down-regulated on day 1 and 3 after challenged. Based on KEGG Pathway enrichment analysis, viral recognition thus appears to be one general aspect of the antiviral response that is not subject miRNA fine-tuning during active IAV infection.

### qPCR validation of RNAseq results

In the present study RNAseq miRNA expression results were validated using high-throughput qPCR. Generally, we found the agreement between RNAseq and qPCR results to be good with respect to directionality of miRNA regulation (up, down, or unchanged). These expression patterns were largely identical in both datasets with only two noticeable exceptions: ssc-miR-339-3p, which was significantly up-regulated in RNAseq but unchanged in qPCR, and mmu-miR-34b-5p, which was significantly down-regulated in RNAseq but also unchanged in qPCR. This lack of agreement could be caused by factors inherent to either method, e.g. poorly designed qPCR primers or the presence of isomiRs which would be detected by RNAseq but not qPCR and would thus contribute to expression levels in one dataset but not the other [72]. However, although there is a general agreement on the regulation patterns of the miRNAs between the two platforms, levels of regulation (i.e. fold change of expression) and statistical significance did not always concur. The relatively small changes that are typically observed when measuring differential miRNA expression combined with considerable animal-to-animal variation and difference in platform sensitivity makes it challenging to produce consistent statistically significant results.

To our knowledge, few studies of large-scale qPCR validation of RNAseq miRNA expression results have yet been published. One such study by Blondal *et al*. described the quantification of miRNAs in serum of hepatitis B and C patients [73], and reported a linear correlation (R^2^) of 0.6054 between fold changes obtained with the two platforms, which is highly comparable to our own findings (R^2^ = 0.6104). Also consistent with our observations is the fact that Blondal *et al*. found the levels of fold change to differ between the two platforms for many of the assayed miRNAs, even though the directionality of regulation was in agreement. Another study of miRNA quantification in the urine of rats with renal tubular injury reported minimal agreement between RNAseq and qPCR results [74]. The authors detected 14 differentially expressed miRNAs with RNAseq and 32 with RT-qPCR; of these, an overlap of only three miRNAs that were supported by both platforms. Our findings as well as the available literature thus suggest that extensive validation of results obtained with RNAseq should be performed with an independent platform, in order to produce consistent and reliable miRNA expression results.

## Supporting information

S1 Appendix Materials and methods. Fully detailed protocols and experimental procedures.
(PDF)

S1 Table Sequences and qPCR efficiencies of primers used in the present study. (XLSX)

S2 Table Comparison of porcine and human miRNA sequences for those applied in miRNA-mRNA interaction analysis. Underlined, bold nucleotides highlights differences between porcine and human sequences. Seed sequences (nucleotides 2–7) are highlighted in upper case.
(XLSX)

S3 Table Details on each sequenced sample regarding read counts and mapping statistics. (XLSX)

S4 Table RNAseq read counts. Normalized read counts for all known porcine (n = 238) as well as potential novel porcine miRNA candidates (n = 15) detected with RNAseq (total n = 253) in 25 porcine lung samples.
(XLSX)

S5 Table Novel porcine miRNA candidate sequences. Mature and hairpin sequences of novel porcine miRNA candidates identified in RNAseq data, for which homologs could be identified in other species.
(XLSX)

S6 Table Expression changes of miRNAs determined by RNAseq. Log_2_FC of all known porcine (n = 238) as well as potential novel porcine miRNA candidates (n = 15) detected with RNAseq (total n = 253) in lungs of pigs at 1, 3, and 14 days after IAV challenge relative to unchallenged controls.
(XLSX)

S7 Table Expression changes of miRNAs measured with qPCR. Log_2_FC of miRNAs determined with qPCR (n = 80) in lungs of pigs at 1, 3, and 14 days after IAV challenge relative to unchallenged controls.
(XLSX)

S8 Table Protein coding genes which were found to be differentially expressed at one or more time points in lungs of pigs after IAV challenge relative to unchallenged controls. Those highlighted in bold are the ones meeting the criteria for being differentially expressed (see text).
(XLSX)

S1 Fig miRNA-target interaction network, up-regulated miRNAs day 1 and 3. Pink edges – experimentally determined interaction. Blue edges – information from curated database. Yellow edges – computationally predicted interaction.
(TIF)

S2 Fig miRNA-target interaction network, down-regulated miRNAs day 1 and 3. Pink edges – experimentally determined interaction. Blue edges – information from curated database. Yellow edges – computationally predicted interaction.
(TIF)
