# Supplementary material for: IFN-λ and microRNAs are important modulators of the pulmonary innate immune response against influenza A (H1N2) infection in pigs
Source: PLoS One. 2018 Apr 20;13(4):e0194765. doi: 10.1371/journal.pone.0194765 (PMC5909910; doi:10.1371/journal.pone.0194765)
Supplement: S1 Appendix — Fully detailed protocols and experimental procedures. (PDF) [file pone.0194765.s001.pdf]

S1 Appendix

Materials and methods

Small RNA sequencing

All procedures related to library preparation, sequencing, and quality control was carried out by the sequencing provider Exiqon A/S (Vedbaek, Denmark). The RNA was diluted to 250 ng/µl in RNase-free water and a total of 8 µl (2 µg RNA) from each sample was supplied to Exiqon A/S for analysis. For each RNA sample, 1 µg of total RNA was converted into miRNA NGS libraries using the NEBNext Small RNA Library Prep Set for Illumina (New England BioLabs) according to the manufacturer's instructions. Each individual RNA sample had adaptors ligated to its 3' (AGATCGGAAGAGCACACGTCTGAACTCCAGTCACNNNNNATCTCGTATGCCGTCTTCTGCTTG) and 5' (AATGATACGGCGACCACCGAGATCACACGTTCAGAGTTCTACAGTCCGACGATC) ends and was converted into cDNA. The cDNA was pre-amplified by PCR (protocol and reagents supplied with NEBNext kit) using the following cycling parameters: initial denaturation at 94 °C for 30 seconds, 15 cycles of denaturation at 94 °C for 15 seconds, annealing at 62 °C for 20 seconds, and extension at 70 °C for 15 seconds, and concluded with final extension at 70 °C for 5 min. The 3' PCR primer was associated with a barcode sequence that was unique for each original RNA sample, thus allowing for differentiation of reads from different samples in the subsequent multiplex sequencing procedure of pooled cDNA samples. Libraries were purified using the QIAquick PCR Purification Kit (Qiagen), and the insert efficiency evaluated on a Bioanalyzer 2100 instrument on high sensitivity DNA chips (Agilent Technologies) to confirm a peak at ~140 nt corresponding to the length of adapter-ligated miRNAs. The miRNA cDNA libraries were size fractionated on a LabChip XT (Caliper Inc.) and a band representing adaptors and 15-40 nt insert (i.e. the size range covering miRNAs) excised according to the manufacturer's instructions. Samples were quantified using qPCR and subsequently pooled in equimolar concentrations. The library pool was used to generate clusters on the surface of a flow cell before sequencing using v3 sequencing methodology in accordance with manufacturer instructions (Illumina). Samples were sequenced on the Illumina NextSeq 500 system, yielding 50 nt single-ended reads of high quality (Q-score above 30). Raw sequencing data were supplied to the authors as compressed FASTQ files.

Small RNA sequencing data analysis

Adaptor sequences from the library preparation and sequencing were trimmed from the reads using Cutadapt v. 1.8 [37] (options applied: -a [3' adapter sequence, see above] -q 20 -m 16) thus retaining only reads with a minimum length of 16 nt. Modules from the miRDeep2 package v. 2.0.0.5 was applied for the remaining sequencing data processing [38]. The mapper module was run to collapse trimmed reads with the following options: -e -h -i -j -m, supplying trimmed sequencing reads in .fq format and specifying an .fa output file with collapsed reads. Bowtie v. 1.0.0 was run with the following options [39]: -p 16 -f -n 1 -e 80 -l 16 -a -m 5 --best –strata [indexed genome file] --sam, using a pre-built indexed porcine genome from the iGenomes collection from Illumina ('susScr3' from UCSC built on the Sscrofa10.2 genome assembly [40], downloaded January 27th 2015 from here: https://support.illumina.com/sequencing/sequencing_software/igenome.html). The miRDeep2 module was supplied with .fa files with trimmed collapsed sequencing reads and the indexed porcine genome (see above), .arf file with reads mapped to the genome, and .fa files with known porcine miRNA sequences and known *Homo sapiens*, *Bos taurus*, and *Mus musculus* miRNA sequences, respectively. The latter was applied for the identification of potential novel (not yet annotated) porcine miRNAs candidates by comparing the obtained read sequences to known mature miRNAs from other species. All miRNA sequences were acquired from miRBase v. 21 [41].

Data was normalized individually for each sample using the number (millions) of mapped reads in that particular sample as normalization factor (performed by the quantifier module contained in miRDeep2). Read counts were obtained from the miRDeep2 output file (.csv), and the remaining analysis for differential expression and statistical analysis was performed in R (v. 3.4.2) [42] using the package edgeR (v. 3.20.1) [43] and applying the general linearized model (glm) approach, testing for significant differential expression between groups using an *F*-test. The Benjamini-Hochberg procedure was applied to control the false discovery rate (FDR) at $\alpha = 0.05$. miRNA regulation was calculated as $\log_2$(fold change) ($\log_2$FC) of expression levels in a post-challenge group relative to the control group. A miRNA was considered to be differentially expressed if it was ≥50 % up- or down-regulated, i.e. $\log_2FC > 0.585$ or $\log_2FC < -0.585$, and significant in an *F*-test ($p < 0.05$).

miRNA RT-qPCR

RNA obtained from IAV infected porcine lung samples were reverse transcribed into cDNA and pre-amplified prior to high-throughput qPCR. 100 ng total RNA was applied in each cDNA synthesis in a reaction volume of 10 µl also containing 1 µM universal RT primer (5' CAGGTCCAGTTTTTTTTTTTTTTTVN 3'), 1 µl 10X poly(A)

polymerase buffer (New England BioLabs), 0.1 mM ATP (New England BioLabs), 0.1 µM of each deoxynucleotide (dATP, dTTP, dGTP, and dCTP) (Sigma-Aldrich), 100 units MuLV reverse transcriptase (replaced with water in –RT control (no reverse transcriptase)) (New England BioLabs), 1 unit poly(A) polymerase (replaced with water in –poly(A) control (no poly(A) polymerase)) (New England BioLabs), and RNase-free water. The cDNA synthesis was carried out at 42 °C for 60 min followed by 95 °C for 5 min. Two cDNA replicates were synthesized from each RNA sample. All cDNA samples were pre-amplified prior to qPCR. Pre-amplification reactions contained 2.5 µl cDNA (diluted 1:10 in low-EDTA TE buffer (VWR – Bie & Berntsen)), 5 µl TaqMan PreAmp Master Mix (Applied Biosystems), and 2.5 µl 200 nM pre-amplification primer pool. The primer pool contained all primers to be included in the subsequent qPCR (at 200 nM each). Pre-amplification parameters were as follows: 95 °C for 10 min followed by 14 cycles of 95 °C for 15 seconds and 60 °C for 4 min. Residual primers were digested by adding 16 units of Exonuclease I (New England BioLabs) at 37 °C for 30 min followed by 80 °C for 15 min. All miRNA primers, including the universal RT primer, were designed as described previously [44] and purchased from Sigma-Aldrich. Whenever a porcine version of a miRNA was and available in miRBase at the time of primer design, this sequence was used (indicated by the prefix ssc). Otherwise, the sequence of the human (hsa) (or mouse, mmu, in the case of novel miRNAs from sequencing data) homolog was used instead. Sequences and qPCR efficiencies for all primers can be found in S1 Table. The majority of miRNAs assayed by RT-qPCR were chosen based on RNAseq results. Additional miRNAs not detected by sequencing were also included in the qPCR analysis, based on our previous work and literature studies. qPCR was carried out in 96.96 Dynamic Array IFC chips using the high-throughput BioMark HD real-time platform (Fluidigm). For each sample, 1.5 µl pre-amplified cDNA (diluted 1:10 in low-EDTA TE buffer (VWR – Bie & Berntsen)) was combined with 3 µl ABI TaqMan Gene Expression Master Mix (Applied Biosystems), 0.3 µl 20X DNA Binding Dye Sample Loading Reagent (Fluidigm), 0.3 µl 20X EvaGreen (Biotium, VWR – Bie & Berntsen), and 0.9 µl low-EDTA TE buffer (VWR – Bie & Berntsen). qPCR primers were prepared by mixing 3 µl primer pair (forward and reverse, 10 µM each) with 3 µl 2X Assay Loading Reagent (Fluidigm). 96 sample mixes (including a non-template control, using water instead of cDNA) and 96 primer mixes were added to the 96.96 Dynamic Array IFC chip, and a total of 9,216 qPCR reactions were carried out in parallel. Dilution series were prepared from a pool of all samples (except –RT and –poly(A) controls) and included in the qPCR to assess primer efficiency. The following thermal protocol was applied: a thermal mix phase consisting of 2 min at 50 °C, 30 min at 70 °C, and 10 min at 25 °C; a hot start phase consisting of 2 min at 50 °C and 10 min at 95 °C; 35 cycles of denaturing for 15 seconds at 95 °C and annealing/elongation for 1 min at 60 °C, with fluorescence being

recorded at the end of each cycle. Melting curve analysis was performed lastly by increasing the temperature form 60 to 95 °C at a speed of 1 °C/3 seconds.

miRNA qPCR data analysis

All amplification and melting curves were visually inspected using the Fluidigm Real-Time PCR Analysis software (v. 4.1.3). Primer efficiency was assessed for each assay from the standard curves produced from the included dilution series and subsequently used to adjust the C_q values in the individual assays. Primer efficiencies ranged from 92 to 119 %. miRNA qPCR data was normalized using the global mean expression method [45] and cDNA replicates were averaged. C_q values were converted to relative quantities on the linear scale. miRNA regulation was calculated as $\log_2$FC of expression levels in a post-challenge group relative to the control group. A miRNA was considered to be differentially expressed if it was ≥50 % up- or down-regulated, i.e. $\log_2 FC > 0.585$ or $\log_2 FC < -0.585$, and significant in a *t*-test ($p < 0.05$, $\log_2$ transformed data), using the T.TEST() function in Microsoft Excel (two-tailed, equal variance).

RT-qPCR of protein coding genes

For mRNA, the cDNA synthesis was performed with 500 ng RNA using the QuantiTect Reverse Transcription Kit (Qiagen). Any residual genomic DNA was removed from the RNA sample by incubating with 2 µl gDNA Wipeout Buffer (Qiagen) in a total reaction volume of 14 µl (in RNase-free water) at 42 °C for 2 min. Next, 6 µl reverse transcription master mix containing 0.75 µl Quantiscript Reverse Transcriptase (replaced with water in –RT control), 1 µl RT Primer Mix (mix of oligo-dT and random primers), 4 µl Quantiscript RT Buffer, and 0.25 µl RNase-free water was added to each sample. cDNA was synthesized at 42 °C for 15 min followed by 95 °C for 3 min. Two cDNA replicates were synthesized from each RNA sample. cDNA was diluted 1:10 in low-EDTA TE buffer prior to pre-amplification and exonuclease treatment, which was carried out as described above (section 'miRNA RT-qPCR') using 16 cycles of amplification. Highly specific mRNA primers were designed according to specification previously described [46] using Primer3 (http://bioinfo.ut.ee/primer3-0.4.0/). Included in the panel of assayed genes were a number of potential reference genes ('housekeeping genes') that would later be evaluated for their suitability for data normalization. Dilution series were prepared from a pool of all samples and included in the qPCR (except the –RT control) to assess primer efficiency. Sequences and qPCR efficiencies for all primers can be found in S1 Table. High-throughput qPCR was carried out on the BioMark HD real-time instrument (Fluidigm) in a setup identical to that described for miRNA (section 'miRNA RT-qPCR').

mRNA qPCR data analysis

All amplification and melting curves were visually inspected using the Fluidigm Real-Time PCR Analysis software (v. 4.1.3). Primer efficiency was assessed from the standard curves used to adjust the C_q values in the individual assays. Primer efficiencies ranged from 95 to 113 %. Potential reference genes were evaluated using the algorithms geNorm [47] and NormFinder [48], and β-actin (*ACTB*), β_2 microglobulin (*B2M*), glyceraldehyde 3-phosphate dehydrogenase (*GAPDH*), peptidylprolyl isomerase A (*PPIA*), 60S ribosomal protein L13A (*RPL13A*), and tyrosine 3-monooxygenase/tryptophan 5-monooxygenase activation protein, zeta polypeptide (*YWHAZ*) were applied for mRNA qPCR data normalization. cDNA replicates were averaged and C_q values were converted to relative quantities on the linear scale. mRNA regulation was calculated as $\log_2$FC of expression levels in a post-challenge group relative to the control group. A protein coding gene was considered to be differentially expressed if it was ≥100 % up- or down-regulated, i.e. $\log_2FC > 1$ or $\log_2FC < -1$, and significant in a *t*-test ($p < 0.05$, $\log_2$ transformed data), using the T.TEST() function in Microsoft Excel (two-tailed, equal variance).

miRNA-mRNA interactions

Using online tools and databases (see below), potential mRNA targets among the assayed protein coding genes were identified for miRNAs found to be differentially expressed after IAV challenge. As very little experimental target validation has been performed for porcine miRNAs, the human miRNA homologs as well as protein coding genes were applied for target identification and prediction. A comparison of porcine and human miRNA sequences applied in miRNA-mRNA interaction analysis can be found in S2 Table, where the seed sequences (nucleotides 2-7) have been highlighted, as these nucleotides are primarily responsible for miRNA targeting specificity. One differentially expressed miRNA was excluded from these analyses, as no human homolog has yet been annotated (ssc-miR-7134-5p, first identified in a previous study of ours [49]). Using each miRNA as input in a 'single name' search in the RAIN database v. 1.0 [50], which integrates non-coding RNA interactions and associations with the protein-protein interaction database STRING v. 10.5 [51], previously experimentally validated and computationally predicted targets were identified by applying the following settings: 'active interaction sources' – only 'experiments' and 'databases'; 'minimum required interaction score' – 'low confidence (0.150)'; 'max number of interactions to show' – custom value of 300 for 1st shell (300 was chosen as this number proved to exceed the final number of nodes in the resulting network for any of the miRNAs, thus ensuring that all possible interactions were shown). As RAIN due to licensing restrictions does not

integrate information from TarBase or target predictions by microT-CDS from DIANA Lab, previously experimentally validated and computationally predicted targets were likewise identified from these two sources. Searches in TarBase v. 7.0 [52] were made using the following settings: 'species' – '*Homo sapiens*'; 'validation type' – 'direct'; 'validated as' – 'positive'. Targets were predicted with microT-CDS v. 5.0 [53] by applying the default score threshold of 0.7. For all validated or predicted miRNA-mRNA interactions obtained from RAIN, TarBase, and microT-CDS, Pearson's correlation coefficient (Pearson's *r*) was calculated for miRNA and mRNA expression data (qPCR) on day 1 and 3 post challenge. If a negative correlation of $r < -0.532$ ($p < 0.05$) was seen, that particular miRNA-mRNA interaction was regarded as potentially relevant in the regulation of innate immune response in pigs during active IAV infection.

The RAIN database v. 1.0 [50] was likewise applied to identify cellular pathways that could potentially be affected by the differentially expressed miRNAs. Two subsets of differentially expressed miRNAs were applied as input in 'multiple names' search in RAIN: 1) miRNAs up-regulated during acute infection on day 1 and/or 3 after challenge and 2) miRNAs down-regulated during acute infection on day 1 and/or 3 after challenge. The following settings were applied to obtain miRNA-target interaction networks in STRING [51]: 'active interaction sources' – only 'experiments' and 'databases'; 'minimum required interaction score' – 'medium confidence (0.400)'; 'max number of interactions to show' – custom value of 300 for 1st shell (300 was chosen as this number proved to exceed the final number of nodes in the resulting network for any of the four sets of miRNAs, thus ensuring that all possible interactions were shown). KEGG Pathway enrichment analysis [54] (integrated in STRING) was performed on the collection of targets identified for each set of the differentially expressed miRNAs.
