# Supplementary figures and images for: IFN-λ and microRNAs are important modulators of the pulmonary innate immune response against influenza A (H1N2) infection in pigs
Source: PLoS One. 2018 Apr 20;13(4):e0194765. doi: 10.1371/journal.pone.0194765 (PMC5909910; doi:10.1371/journal.pone.0194765)

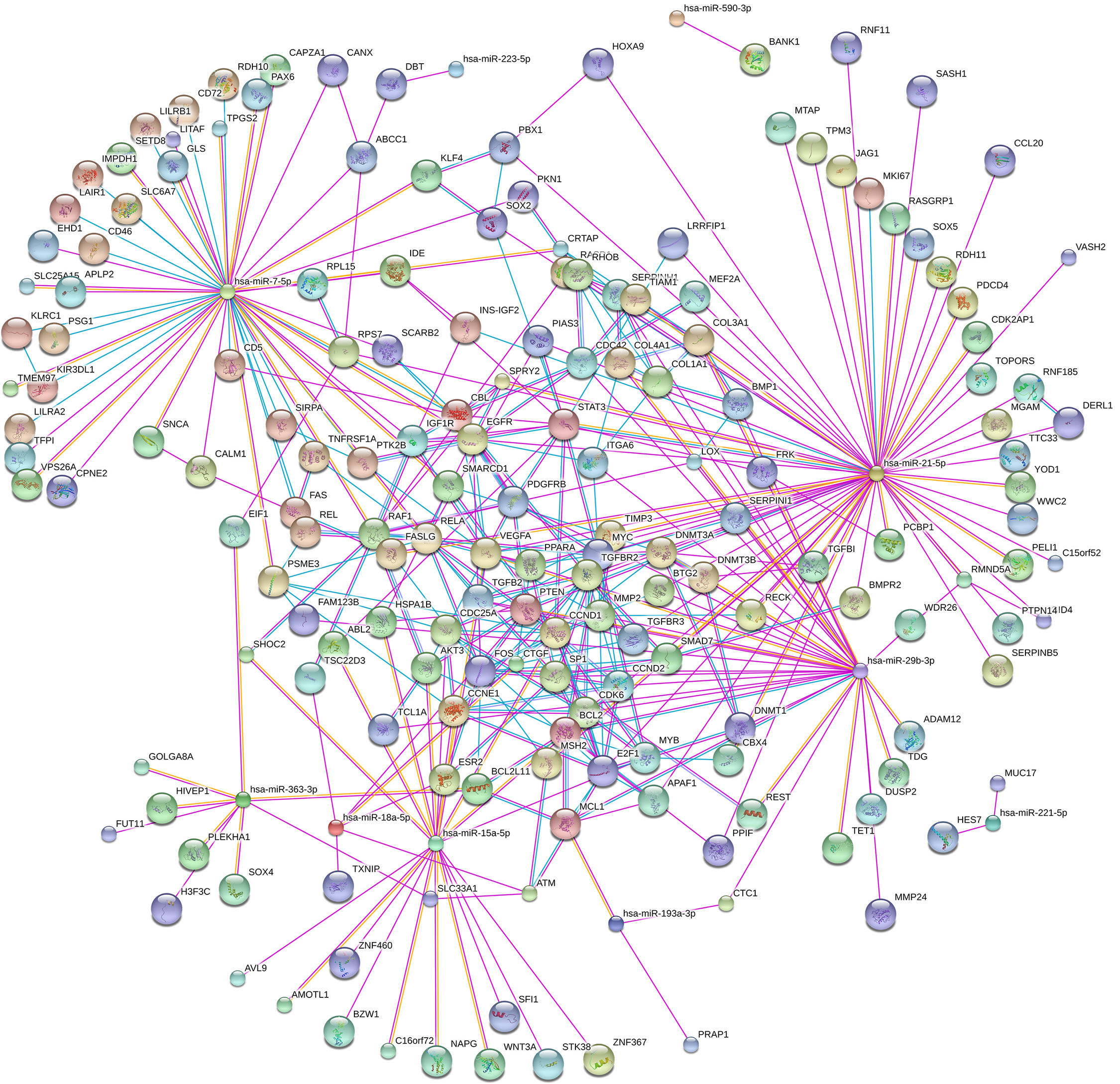

Supplement: S1 Fig — Pink edges – experimentally determined interaction. Blue edges – information from curated database. Yellow edges – computationally predicted interaction. (TIF) [file pone.0194765.s010.tif]

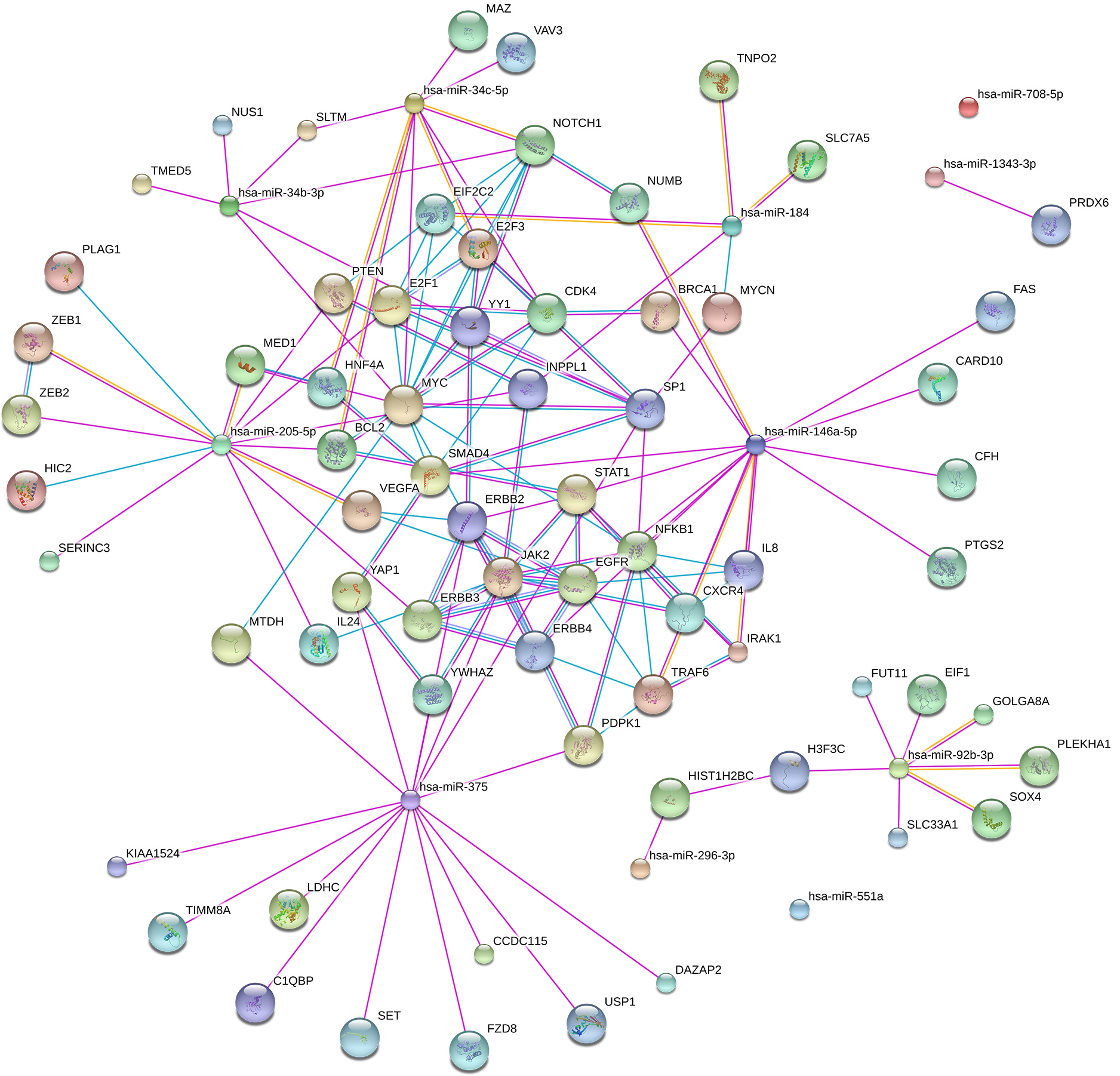

Supplement: S2 Fig — Pink edges – experimentally determined interaction. Blue edges – information from curated database. Yellow edges – computationally predicted interaction. (TIF) [file pone.0194765.s011.tif]
